# Targeting *Mycobacterium tuberculosis* CoaBC through Chemical Inhibition of 4′-Phosphopantothenoyl-l-cysteine Synthetase (CoaB) Activity

**DOI:** 10.1021/acsinfecdis.0c00904

**Published:** 2021-05-03

**Authors:** Joanna C. Evans, Dinakaran Murugesan, John M. Post, Vitor Mendes, Zhe Wang, Navid Nahiyaan, Sasha L. Lynch, Stephen Thompson, Simon R. Green, Peter C. Ray, Jeannine Hess, Christina Spry, Anthony G. Coyne, Chris Abell, Helena I. M. Boshoff, Paul G. Wyatt, Kyu Y. Rhee, Tom L. Blundell, Clifton E. Barry, Valerie Mizrahi

**Affiliations:** † MRC/NHLS/UCT Molecular Mycobacteriology Research Unit & DST/NRF Centre of Excellence for Biomedical TB Research & Wellcome Centre for Infectious Diseases Research in Africa, Institute of Infectious Disease and Molecular Medicine and Department of Pathology, Faculty of Health Sciences, University of Cape Town, Anzio Road, Observatory 7925, South Africa; ‡ Drug Discovery Unit, College of Life Sciences, University of Dundee, Dow Street, Dundee DD1 5EH, Scotland, U.K.; § Department of Biochemistry, University of Cambridge, 80 Tennis Court Road, Cambridge CB2 1GA, U.K.; ∥ Department of Microbiology and Immunology, Weill Cornell Medical College, New York, New York 10065, United States; ⊥ Yusuf Hamied Department of Chemistry, University of Cambridge, Lensfield Road, Cambridge CB2 1EW, U.K.; # Tuberculosis Research Section, Laboratory of Clinical Immunology and Microbiology, National Institute of Allergy and Infectious Disease, National Institutes of Health, 9000 Rockville Pike, Bethesda, Maryland 20892, United States

**Keywords:** tuberculosis, drug discovery, coenzyme A, CoaBC

## Abstract

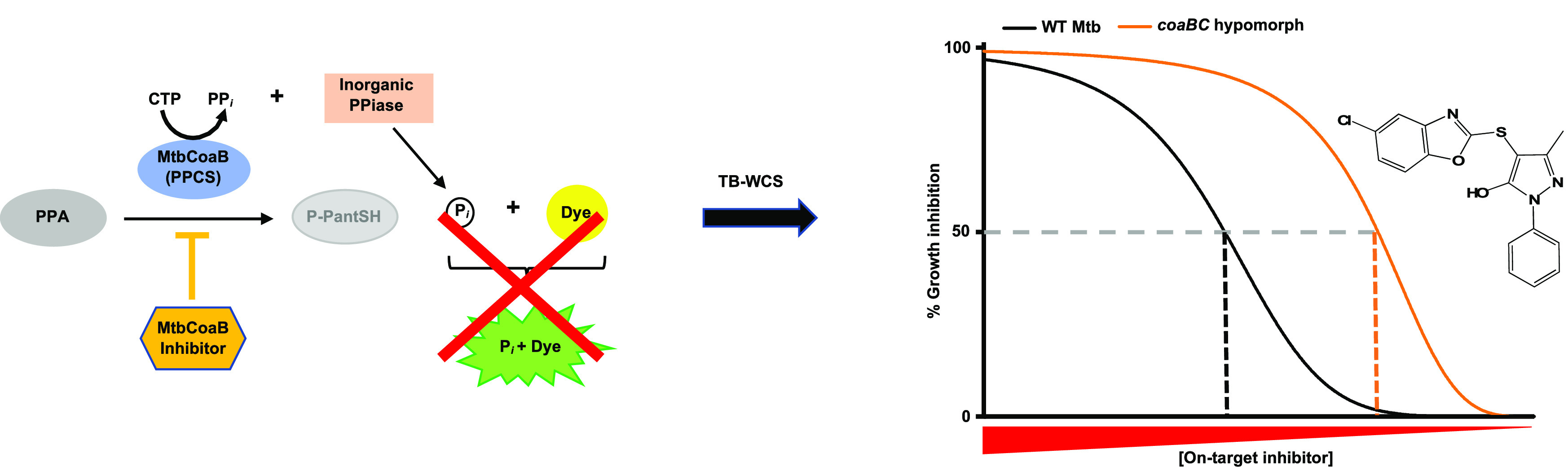

Coenzyme A (CoA) is a ubiquitous cofactor present in all living cells and estimated to be required for up to 9% of intracellular enzymatic reactions. *Mycobacterium tuberculosis* (Mtb) relies on its own ability to biosynthesize CoA to meet the needs of the myriad enzymatic reactions that depend on this cofactor for activity. As such, the pathway to CoA biosynthesis is recognized as a potential source of novel tuberculosis drug targets. In prior work, we genetically validated CoaBC as a bactericidal drug target in Mtb *in vitro* and *in vivo*. Here, we describe the identification of compound **1f**, a small molecule inhibitor of the 4′-phosphopantothenoyl-l-cysteine synthetase (PPCS; CoaB) domain of the bifunctional Mtb CoaBC, and show that this compound displays on-target activity in Mtb. Compound **1f** was found to inhibit CoaBC uncompetitively with respect to 4′-phosphopantothenate, the substrate for the CoaB-catalyzed reaction. Furthermore, metabolomic profiling of wild-type Mtb H37Rv following exposure to compound **1f** produced a signature consistent with perturbations in pantothenate and CoA biosynthesis. As the first report of a direct small molecule inhibitor of Mtb CoaBC displaying target-selective whole-cell activity, this study confirms the druggability of CoaBC and chemically validates this target.

Tuberculosis (TB) remains the leading cause of death from an infectious disease worldwide, claiming an estimated 1.4 million lives in 2019.^1^ The high disease burden coupled with the ongoing emergence and spread of strains of *Mycobacterium tuberculosis* (Mtb) resistant to first- and second-line TB drugs underscores the urgent need to develop new antitubercular agents for the treatment of both drug-resistant and drug-susceptible forms of the disease.^2,3^ In recent years, considerable progress has been made in developing a TB drug pipeline,^4^ which has begun to deliver promising new drugs and drug combinations.^3,5−8^ However, as attrition rates are high across the entire TB drug pipeline there is an urgent need for replenishment of the pipeline, starting from the earliest stage of hit identification. This need is driving drug discovery efforts, which are aimed at delivering high-quality hit compounds with novel mechanisms of action.^9−14^

The coenzyme A (CoA) biosynthetic pathway has attracted attention as a source of new drug targets in a number of bacterial pathogens,^15−20^ including Mtb,^21^ owing to its ubiquity and the lack of sequence similarity between prokaryotic CoA biosynthesis enzymes and their eukaryotic counterparts.^22^ CoA serves as a cofactor for key metabolic enzymes involved in numerous cellular pathways, with 9% of all enzymes estimated as being dependent on this essential cofactor.^18,23^ Of particular significance in the case of Mtb is the critical role played by CoA in the biosynthesis of lipids,^21^ which comprise essential components of the cell envelope, and virulence factors,^24^ as well as in the catabolism of lipids, which provides the primary source of energy for the bacillus during infection.^25^ The availability of crystal structures of a number of prokaryotic CoA pathway enzymes has enabled structure-guided inhibitor design.^26−29^ However, although potent direct inhibitors of Mtb pantothenate synthetase (PanC, *Rv3602c*) and pantothenate kinase (PanK, CoaA, *Rv1092c*) have been developed using this approach, these have failed to translate into lead compounds with significant whole-cell activity against Mtb.^29−32^

The synthesis of CoA from its natural precursor, pantothenate (vitamin B_5_, Pan), occurs in five universal enzymatic reactions, the second and third of which are carried out by a bifunctional CoaBC enzyme encoded by a single gene in most bacteria, including Mtb (*Rv1391*).^33^ This enzyme comprises a C-terminal CoaB domain with 4′-phosphopantothenoyl-l-cysteine synthetase (PPCS) activity and an N-terminal CoaC domain with 4′-phosphopantothenoyl-l-cysteine decarboxylase (PPCDC) activity, which together catalyze the conversion of 4′-phosphopantothenate (PPA) into 4′-phosphopantetheine (P-PantSH)^34^ (Figure 1). The reaction occurs in three steps, the first two of which are carried out by CoaB to produce a 4′-phosphopantothenoyl-l-cysteine intermediate,^35^ which is subsequently decarboxylated at the cysteine moiety by CoaC to produce P-PantSH.^36^ In prior work, we genetically validated CoaBC as a drug target by demonstrating that *coaBC* silencing is bactericidal in Mtb *in vitro* as well as during both the acute and chronic stages of infection in mice.^33^ However, reports of potent and selective small molecule inhibitors of bacterial CoaBC enzymes are scarce, and those compounds identified have largely failed to display significant whole-cell activity.^37,38^ Importantly, the druggability of prokaryotic CoaBC enzymes was established by the identification of the natural product CJ-15,801, which acts as the precursor to a tight-binding CoaB (PPCS) inhibitor with potent activity against *Staphylococcus aureus*.^19^ Furthermore, the recent identification of a small molecule inhibitor of Mtb phosphopantetheinyl transferase (PptT),^24^ required for the transfer of the P-PantSH moiety of CoA to acyl carrier proteins (ACPs) involved in lipid production in Mtb, and the identification of PanD as the target of the bioactive form of pyrazinamide^39,40^ have validated the utility of key enzymes involved in CoA metabolism as antitubercular drug targets.

**Figure 1:**
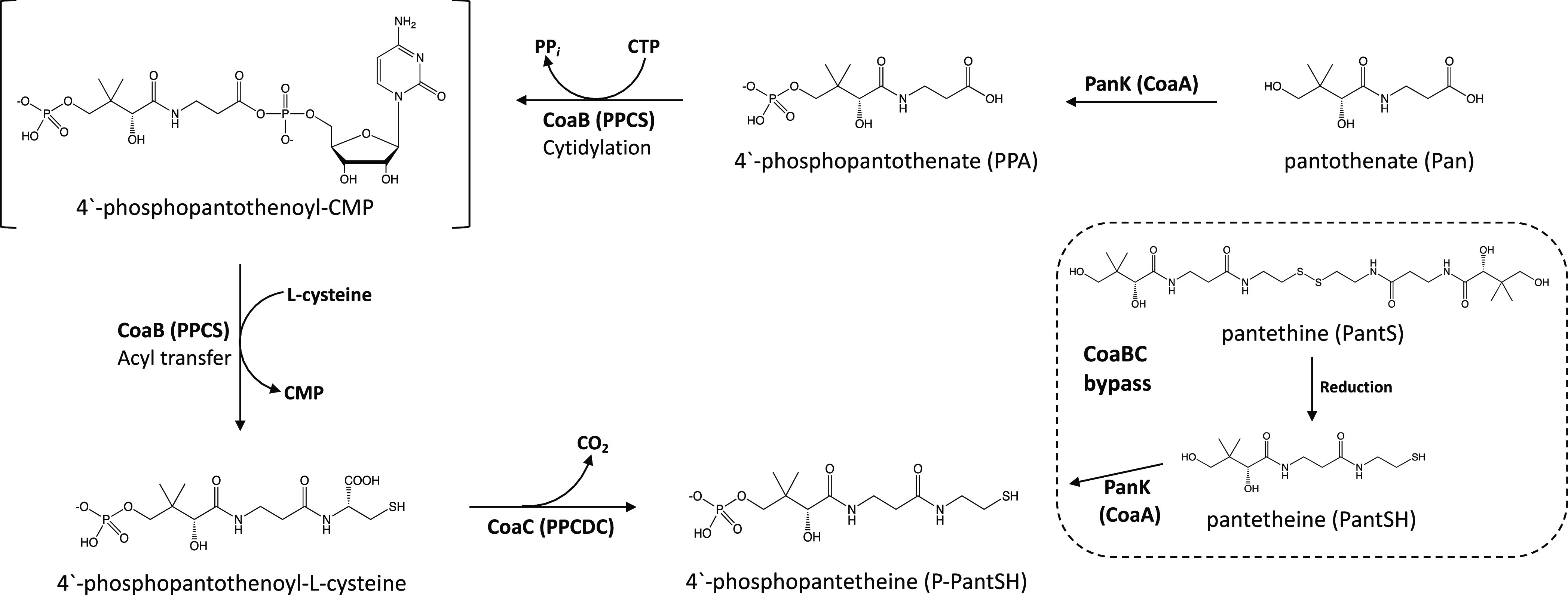
Reaction catalyzed by CoaBC in Mtb. The CoaB domain of the bifunctional protein converts 4′-phosphopantothenate (PPA) into 4′-phosphopantothenoyl-l-cysteine in two steps via the production of a 4′-phosphopantothenoyl-CMP intermediate. Decarboxylation of 4′-phosphopantothenoyl-l-cysteine by the CoaC domain results in the production of 4′-phosphopantetheine (P-PantSH). CoaBC bypass occurs via PanK-mediated phosphorylation of pantetheine (PantSH) to produce P-PantSH. PPCS, phosphopantothenoyl-l-cysteine synthetase; PPCDC, phosphopantothenoyl-l-cysteine decarboxylase.

We recently reported the first crystal structure of the mycobacterial CoaBC together with the first potent and selective inhibitors of Mtb CoaB.^41^ However, these potent inhibitors of CoaB activity displayed limited whole-cell activity against Mtb due to impaired permeability/efflux and intracellular metabolism.^41^ In this study, we confirm the druggability of Mtb CoaBC by identifying a new CoaB inhibitor that confers whole-cell activity against Mtb and providing evidence linking the growth inhibitory effects of this compound on Mtb to inhibition of CoaBC.

## Results

### High-Throughput Screening to Identify Inhibitors of Mycobacterial CoaB

High-throughput screening of 215 000 small molecules from the DDU compound library was carried out to identify inhibitors of Mtb CoaB activity using an adaptation of the BIOLMOL Green end-point pyrophosphate quantification assay, as previously described.^41^ This compound library predominantly comprises commercially available molecules representing a wide range of chemical space that possessing the favorable physicochemical and molecular properties required for a potential preclinical drug candidate, with a small subset of the library (<0.5%) comprising proprietary compounds with known phenotypic activity against a number of various pathogens. The primary screen, which had an overall hit rate of 0.6%, led to the identification of compound **1a**, with an IC_50_ of 9.9 μM against Mtb CoaB. Following confirmation of the target-specific inhibitory activity of **1a** by demonstration of its inactivity in a counter-screen designed to eliminate false positive hits arising due to inactivation of the pyrophosphatase, a further 8 series analogues were synthesized, with the initial structure–activity relationship (SAR) investigation focused on probing the pharmacophore to understand the impact of the *N*-phenyl and *S*-phenyl substituents on their Mtb CoaB activity (Table 1). Replacement of the *S*-phenyl group of **1a** with either 4-methylphenyl (**1b**), 4-chlorophenyl (**1c**), or 2,4-dichlorophenyl (**1d**) led to modest improvements in activity against Mtb CoaB, while introduction of 4-methoxyphenyl (**1e**) led to a 4-fold increase in potency. Conversely, introduction of a substituted benzoxazole (**1f**) led to a modest reduction in potency against Mtb CoaB (Table 1). Introduction of either a 3-chlorophenyl (**1h**) or 4-chlorophenyl (**1g**) at the *N1*-position was tolerated with respect to Mtb CoaB activity, while introduction of a chloropyridine at this position (**1i**) led to a complete loss of inhibitory activity (Table 1).

**Table 1:**
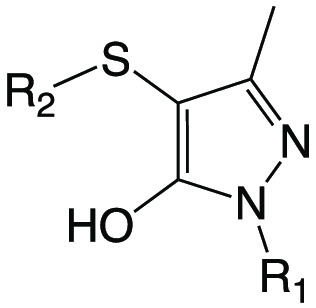

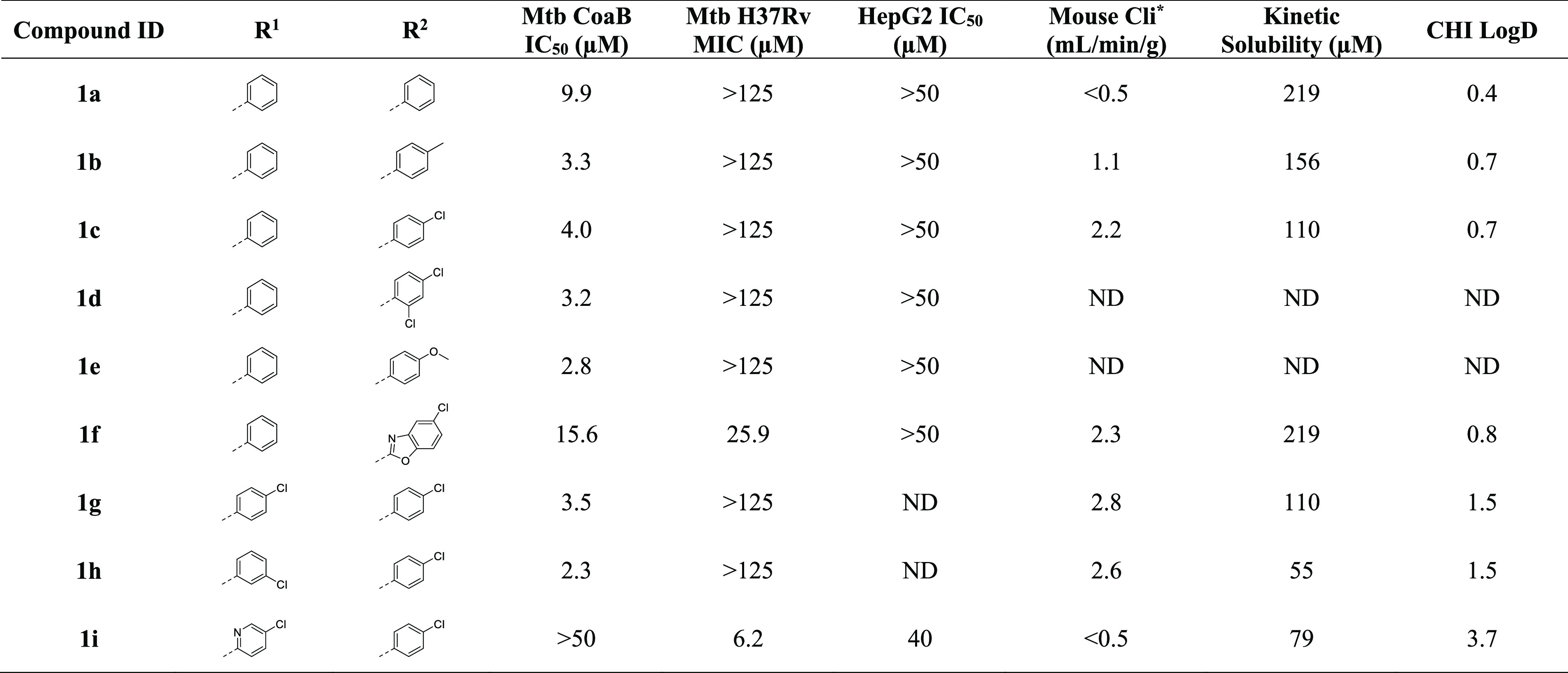
SAR and *In Vitro* Profiling of Modifications to Compound **1a**^a^

a Intrinsic clearance (Cli) using CD1 mouse liver microsomes. ND = not determined. CHI, chromatographic hydrophobicity index.

Assessment of the pharmacological properties of the compounds revealed that none were overly lipophilic, with all except **1i** having CHI LogD values ≤1.5, and their kinetic solubility limits ranged between 55 and 219 μM. The compounds showed good stability in mouse liver microsomes, all having clearance values of ≤2.8 (mL/min)/g and therefore meeting our internal requirement of <5 (mL/min)/g for progression of an early hit compound. None of the compounds showed significant cytotoxicity against HepG2 cells (IC_50_ > 50 μM) with the exception of **1i**, which showed modest activity (IC_50_ = 40 μM).

### Whole-Cell Screening of Series Analogues against Mtb *In Vitro*

Minimum inhibitory concentrations (MICs) of the 9 compounds were determined for wild-type Mtb (strain H37RvMA)^42^ grown in Middlebrook 7H9 media supplemented with albumin-dextrose-catalase (ADC) and glycerol. With the exception of **1f** and **1i**, none of the compounds displayed whole-cell activity against replicating Mtb at concentrations up to 125 μM (Table 1). Although the introduction of a substituted benzoxazole did not substantially affect the activity of **1f** in the biochemical assay, this modification resulted in a modest improvement in whole-cell activity against Mtb (MIC = 25.9 μM) (Table 1). The introduction of a chloropyridine at the *N1*-position in **1i** led to complete ablation of activity in the biochemical CoaB assay; interestingly, this resulted in significantly improved activity against Mtb (MIC = 6.2 μM), thus implicating another target(s) in the mechanism of antimycobacterial action of this analogue.

### Assessment of Target Selectivity in Mtb Using a *coaBC* Hypomorph

**Figure 2:**
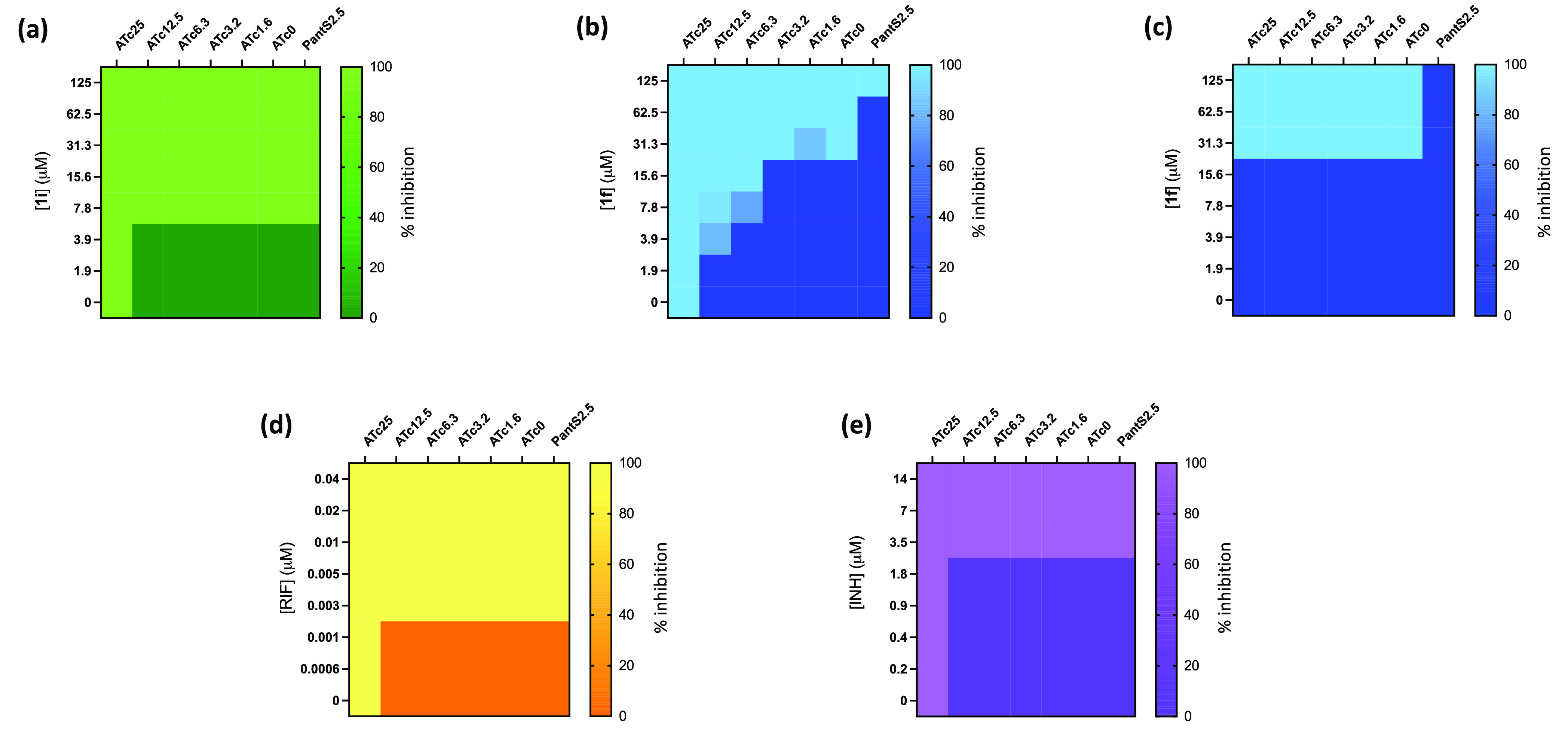
Target-based whole-cell screening of compounds **1i** and **1f** against a *coaBC* Tet-OFF hypomorph. The impact of ATc-mediated transcriptional silencing of *coaBC* on the relative growth of a *coaBC* hypomorph (a,b; d,e) and wild-type Mtb H37RvMA (c) in the presence of increasing concentrations of **1i** (a), **1f** (b, c), rifampicin (d), and isoniazid (e) was determined. Data are representative of the mean and SD of independent triplicates. ATc, anhydrotetracycline; Tet-OFF, mutant whose growth is inhibited in the presence of ATc; PantS2.5, pantethine (2.5 mg/mL); RIF, rifampicin; INH, isoniazid.

As reported previously, we and others have demonstrated the utility of conditional knockdown mutants (hypomorphs) as tools to ascertain the target selectivity of inhibitors in Mtb by means of target-based whole-cell screening.^43−48^ This approach entails assessing whether cellular depletion of the target of interest increases the potency of compounds with biochemical activity against that target. The parent compound and all analogues were subjected to screening in checkerboard assays using a *coaBC* Tet-OFF hypomorph^33^ in which *coaBC* expression is under control of a tetracycline-regulated promoter such that progressive transcriptional silencing of *coaBC* occurs upon addition of increasing concentrations of the inducer, anhydrotetracycline (ATc). The presence of ATc had no discernible effect on the potency of 8 of the 9 compounds against the *coaBC* hypomorph at inhibitor concentrations up to 125 μM. While the lack of hypersensitization of the hypomorph in the case of compound **1i** (Figure 2a) is expected given its lack of biochemical activity against CoaB, the lack of hypersensitization of the hypomorph to **1a**–**1e**, **1g**, and **1h** suggests that the inability of these compounds to inhibit growth of Mtb is likely attributable to compound permeation, efflux, and/or metabolism. In contrast, progressive transcriptional silencing of *coaBC* in the presence of increasing concentrations of ATc induced dose-dependent hypersensitization of the *coaBC* hypomorph to **1f**, resulting in a ∼7-fold decrease in MIC in the presence of 12.5 ng/mL ATc, an observation indicative of on-target activity in Mtb (Figure 2b). Conversely, ATc had no effect on the activity of **1f** against wild-type Mtb H37RvMA (Figure 2c), which had a MIC equivalent to that observed for the *coaBC* hypomorph at low ATc concentrations and in its absence (0–3.2 ng/mL) (Figure 2b). This result is consistent with the fact that the level of *coaBC* expression in the *coaBC* Tet-OFF hypomorph in the absence of ATc is similar to that observed in wild-type Mtb.^33^ Moreover, ATc did not render the *coaBC* hypomorph more susceptible to the first-line antitubercular drugs rifampicin and isoniazid (Figure 2d,e) whose mechanisms of action are unrelated to CoA biosynthesis or utilization. Therefore, *coaBC* transcriptional silencing selectively renders Mtb hypersensitive to **1f**, suggesting that this compound mediates growth inhibition of Mtb, at least in part, via inhibition of CoaBC activity.

### Confirmation of the Target Selectivity of Compound **1f** in Mtb by CoaBC Bypass

We previously confirmed the functionality of CoaBC bypass in Mtb via phosphorylation of pantetheine (PantSH), the reduced form of pantethine (PantS), by the Type I PanK (CoaA) to produce P-PantSH, the product of the CoaBC-catalyzed reaction (Figure 1).^33,37,49^ It therefore follows that if **1f** is acting by inhibition of CoaBC supplementation of the growth medium with exogenous PantS should rescue Mtb from the inhibitory effects of this compound. Indeed, supplementation with PantS rescued the wild-type strain from **1f** toxicity (Figure 2c) and led to a 4-fold reduction in susceptibility of the *coaBC* hypomorph to **1f**, although rescue of the hypomorph was not observed at the highest concentration of **1f** tested (Figure 2b). To exclude the possibility that conversion of PantS to Pan via hydrolysis could result in increased production of PPA, which might in turn increase the activity of CoaBC and thereby render **1f** less active against the *coaBC* hypomorph, we assessed the ability of Pan to rescue growth of this strain under conditions of maximal *coaBC* silencing (i.e., at an ATc concentration of 200 ng/mL). Although Pan fully rescued Mtb from the growth inhibitory effects of PanC deficiency in a *panC* Tet-OFF hypomorph,^43^ as expected, it was unable to restore growth of the *coaBC* hypomorph (Figure S1). This observation argues against the existence of pantetheinase (vanin) activity (enzymes that catalyze the hydrolysis of PantSH to Pan and are abundantly present in mammalian tissues and serum^50^) in Mtb and, thus, against a confounding role for PantS hydrolysis and subsequent alteration in the physiological flux through the canonical pathway in the PantS-mediated rescue of Mtb from **1f** toxicity.

Given the propensity of Mtb hypomorphs to acquire suppressor mutations that abrogate their ATc responsiveness,^44^ especially upon prolonged propagation and/or serial passage, the *coaBC* hypomorph was grown to a lower cell density (OD_600_) than the wild-type comparator for the use in the MIC assays shown in Figure 2. We therefore hypothesized that the discrepancy in PantS rescue observed between the wild-type and *coaBC* hypomorph strains at the highest concentration of **1f** tested (125 μM) could be due to an inoculum effect. Consistent with this hypothesis, the MICs of **1f** in the presence of PantS (2.5 mg/mL) were found to be 83.3 μM and 77.2 μM for the wild-type and the *coaBC* hypomorph, respectively, when the cultures used to inoculate the MIC plates were at an OD_600_ of 0.2, but increased to >125 μM when the inoculating cultures were at a higher cell density (OD_600_ = 0.6). Importantly, the inhibitory activity of **1f** against the wild-type strain was unaffected by the density of inoculating culture (MIC = 25.9 μM, assessed at OD_600_ = 0.2 or 0.6), and the presence of ATc did not confer any non-specific growth inhibitory effects at the lower cell density (Figure S2). Taken together with the ATc dose-dependent hypersensitization of the *coaBC* hypomorph, these results support the conclusion that the antibacterial activity of **1f** is mediated through inhibition of CoaBC.

### Elucidation of the Mode of Inhibition and Selectivity of Compound **1f**

**Figure 3:**
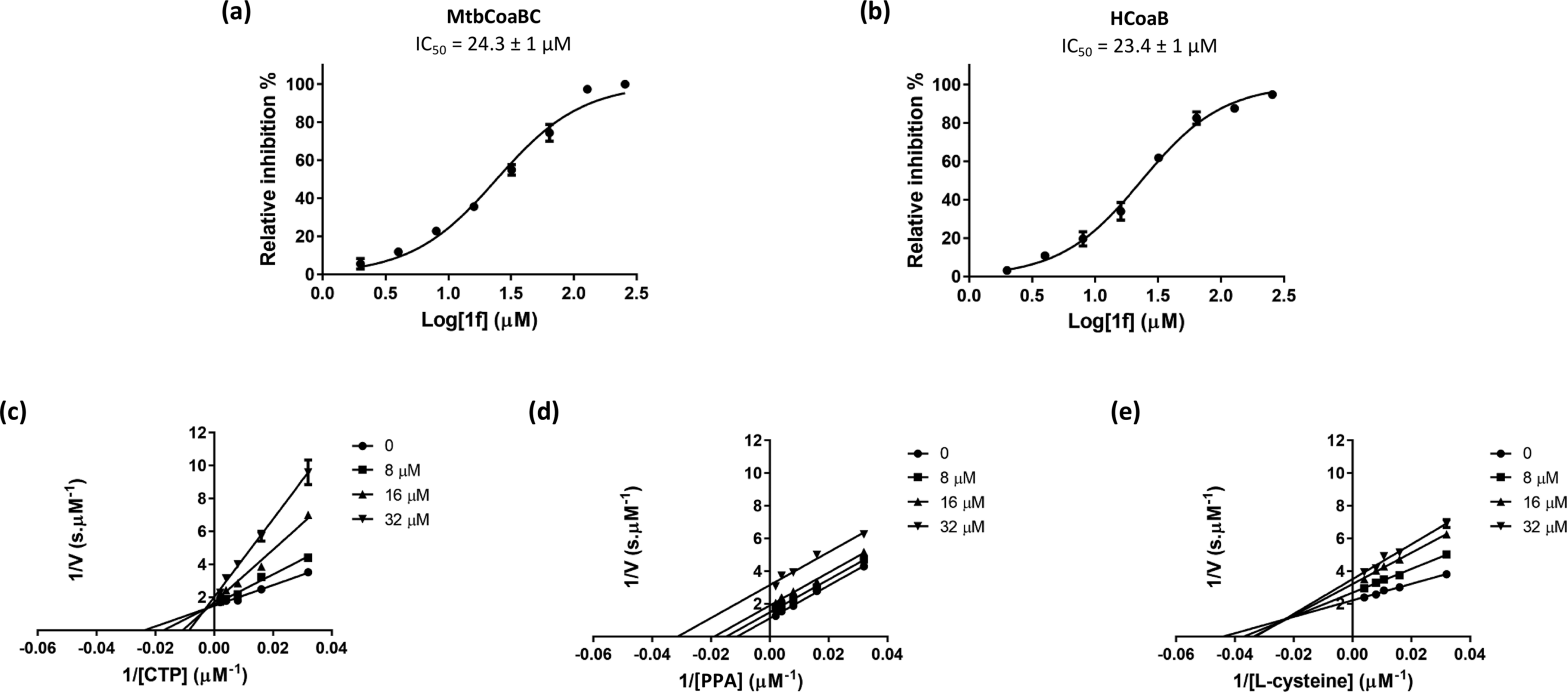
CoaBC inhibition by compound **1f**. Dose response profile for MtbCoaBC (a) and HCoaB (b). Lineweaver–Burk plots showing the effect of varying concentrations of compound **1f** in the presence of varying concentrations of CTP (c), PPA (d), and l-cysteine (e). Data are representative of the mean and SD of independent triplicates.

To assess the mode of inhibition of CoaBC by **1f**, competition experiments were carried out between the compound and the three substrates of CoaB (PPA, CTP, and l-cysteine) using the EnzChek coupled enzyme assay, as previously described.^41^ We first confirmed that compound **1f** was inactive on the coupled reporter enzymes at 100 μM. The IC_50_ for this compound using the EnzChek assay was determined to be 24.3 μM against the Mtb CoaBC, comparable to that observed in the high-throughput screen (Figure 3a). To evaluate the selectivity of compound **1f**, the Mtb enzyme was substituted with human CoaB in the EnzChek coupled enzyme assay. A very similar dose–response profile was observed with an IC_50_ value of 23.4 μM, suggesting poor selectivity of this compound for the Mtb enzyme (Figure 3b).

Mtb CoaB exhibited an apparent *K*_m_ of 27.4 ± 2.6, 45.4 ± 5.3, and 29.8 ± 3.0 μM for CTP, PPA, and l-cysteine, respectively (Figure S3). Inhibition experiments demonstrated that **1f** displays uncompetitive inhibition for PPA, with an α*K*_i_ of 19.0 ± 0.6 μM, and noncompetitive inhibition for CTP and l-cysteine, with *K*_i_ values of 10.1 ± 1.4 μM and 22.7 ± 7.6 μM, respectively (Figure 3c–e), consistent with the compound binding preferentially in the presence of PPA. This is in contrast to our previous observation with allosteric inhibitors of CoaBC, which bound preferentially in the presence of all three substrates.^41^ We therefore decided to employ a different approach to further validate these findings. Using differential scanning fluorimetry (DSF), the melting temperature of CoaBC in the absence of natural ligands was determined to be 46 °C (Table 2; Figure S4). Evaluation of the effect of **1f** on the melting temperature of CoaBC in the presence of the three substrates resulted in a substantial shift in melting temperature only when PPA was present, regardless of the presence or absence of other substrates and products (Table 2), thus further confirming that binding of compound **1f** to CoaB is dependent on the presence of PPA (Table 2). The negative melting shift observed in the presence of PPA and l-cysteine is consistent with a mixed inhibition profile for l-cysteine.

**Table 2:** Effect of Compound **1f** on the Melting Temperature of Mtb CoaBC in the Presence of the CoaB Substrates CTP, PPA, and l-Cysteine

	Thermal shift relative to CoaBC^a^ (°C)		
Substrates (1 mM)	Substrate	Substrate + **1f** (2.5 mM)	Δ*T*_m_^b^
No substrate	–	1.5	
CTP	5.5	5.5	0
CMP	1.5	2.5	1
PPA	3.5	10.5	7
l-cysteine	0.5	1.5	1
CMP + l-cysteine	1.5	2.5	1
CMP + PPA	4.5	10.5	7
CMP + l-cysteine + PPA	4.5	11	6.5
l-cysteine + PPA	4.5	1.5	–3
CTP + PPA	11.8 ± 0.8	18.8 ± 0.3	7
CTP + l-cysteine	5.2 ± 0.3	5.2 ± 0.3	0

a CoaBC melting temperature under the assay conditions used is 46 °C. Data are representative of three independent triplicates. Where SD is not indicated, SD = 0.

b Δ*T*_m_ represents the difference in melting temperature in the presence and absence of compound **1f** under each condition.

Although it was previously reported that binding of PPA to *Enterococcus faecalis* CoaB occurs after CTP binding,^51^ the ability of PPA alone to substantially shift the melting temperature of Mtb CoaBC suggests that PPA is able to bind to the Mtb CoaB in the absence of CTP (Table 2). In *Escherichia coli*, binding of PPA to CoaB causes movements in the protein that close the PPA binding site, thereby limiting access to the CTP binding site^52^ (Figure S5). Mechanistically, this triggers the formation of a dead-end complex in which PPA and compound **1f** bind to the free enzyme, preventing CTP binding and thereby blocking catalysis. This accounts for the inhibition profile of compound **1f**, which is very close to competitive inhibition in the case of CTP. A crystal structure of CoaB with **1f** bound could not be obtained, but the observed mode of inhibition together with known movements that occur in the protein after PPA binding (Figure S5), with a flexible loop covering the PPA binding site upon binding of this substrate,^52^ suggests a different binding mode for **1f** with distinct properties from the previously reported allosteric inhibitors.^41^

### Metabolic Consequences of Exposure of Mtb to Compound **1f**

**Figure 4:**
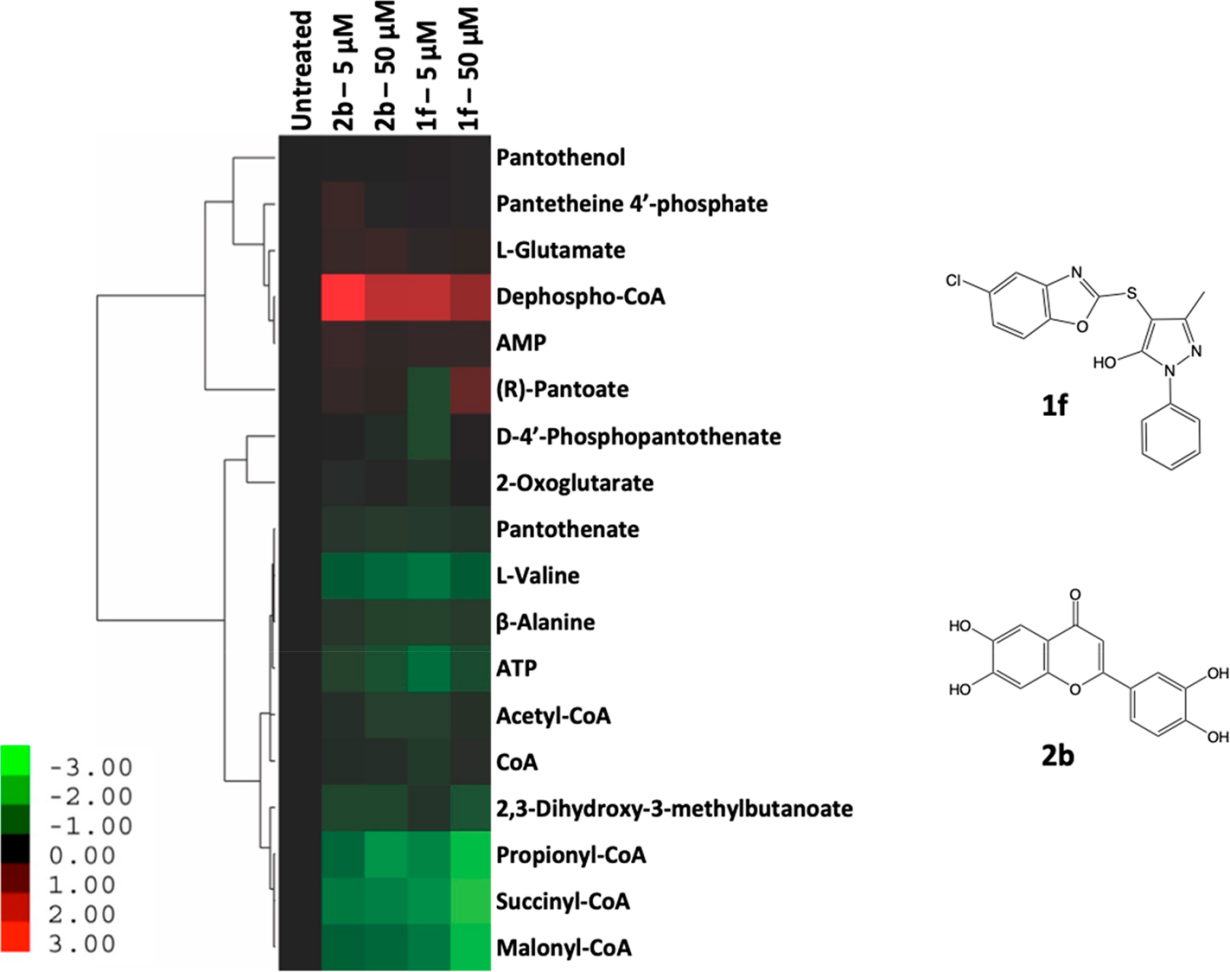
Metabolic impact of exposure of wild-type Mtb H37RvMA to **1f** and **2b** at 5 μM and 50 μM. Relative metabolite abundances (based on ion intensities) are indicated in heat map format, where relative abundance refers to the change in the abundance of a given metabolite in the presence of varying concentrations of **1f** and **2b** as compared to an untreated (DMSO) control. Data are log2 transformed, with columns representing individual treatments, as indicated, and vertical clustering by rows revealing groups of metabolites exhibiting similar responses to drug exposure. Data are representative of independent triplicates and were parsed by uncentered Pearson’s correlation with centroid linkage clustering and rendered using the image generation program Java TreeView (http://jtreeview.sourceforge.net/).

The metabolic derangement resulting from exposure of Mtb to 5 μM or 50 μM of either **1f** or the potent allosteric CoaB inhibitor **2b** (IC_50_ = 0.08 μM)^41^ (Figure 4) for 24 h was determined, as previously described.^53^ Unlike **1f**, which is active against Mtb (MIC = 25.9 μM), compound **2b** did not display significant whole-cell activity (MIC > 250 μM).^41^ Interestingly, however, both compounds elicited similar alterations in the abundance of metabolites specifically associated with Pan and CoA biosynthesis as compared to untreated cells (Figure 4; Figure S6). Exposure to both compounds produced a distinct metabolomic signature involving metabolites related to the CoA pathway, which was largely consistent with that observed following transcriptional silencing of *coaBC* in Mtb.^33^ Specifically, there was substantial accumulation of dephospho-CoA, the immediate precursor of CoA, and notable depletion of the CoA thioesters propionyl-CoA, succinyl-CoA, and malonyl-CoA relative to untreated bacilli (Figure 4; Figure S6). The latter effect, which was dose-dependent in the case of **1f**, is consistent with the reduced levels of malonyl- and acetyl-CoA observed upon transcriptional silencing of *coaBC*.^33^ Unlike the CoA depletion observed upon *coaBC* silencing,^33^ no detectable change in the level of CoA was observed in Mtb treated with either compound **1f** or **2b** under the conditions tested (Figure 4). Notably, however, while analogous alterations in the levels of all other metabolites were observed after 1.5 days of *coaBC* silencing, depletion of CoA was only observed in the *coaBC* hypomorph at day 3. This may reflect the longevity of this cofactor in Mtb, mediated by the reassembly of CoA via recycling of its P-PantSH moiety from holo-ACPs in the presence of ATP, as has been observed in *E. coli*.^54^

Finally, the fate of the compounds upon exposure to Mtb was followed by monitoring their intracellular accumulation. Both **1f** and **2b** were found to accumulate intracellularly in Mtb in a dose-dependent manner, but the intracellular abundance of **1f** was substantially greater than **2b** at both concentrations tested (Figure S7). To exclude the possibility that this could be due to inherent or bacterial-induced differences in the ionization efficiencies of these compounds, standard curves of **1f** and **2b** spiked into bacterial extracts were generated. The comparable ionization efficiencies observed for both compounds (Figure S8) further support our observation that the intracellular abundances of these compounds vary substantially, corroborating our previous observation that the phenotypic divergence between these compounds is likely due to the rapid biotransformation of **2b**.^41,55,56^ It is therefore reasonable to conclude that the comparatively low intracellular level to which compound **2b** accumulates in Mtb over 24 h is sufficient to induce metabolomic perturbations but does not reach the threshold level required to manifest in a growth phenotype through inhibition of CoaBC.

## Discussion

CoA is a ubiquitous cofactor across all domains of life. The sequence divergence between prokaryotic and eukaryotic components of the CoA biosynthesis pathway^22^ makes it a potentially attractive source of novel drug targets in pathogens that include Mtb.^21^ This notion has been reinforced by the identification of PanD as the target of pyrazinoic acid, the bioactive form of the first-line antitubercular drug pyrazinamide,^39,40^ and the discovery of an inhibitor of Mtb PanK requiring activation by the monooxygenase, EthA.^57^ However, efforts to identify inhibitors of enzymes in the CoA pathway of Mtb by target-based approaches have largely stalled at the level of whole-cell activity. Using hypomorphs of Mtb as tools to assess the target selectivity of small-molecule inhibitors of PanC and PanK, we^43^ and others^31^ concluded that these failures might be explained, at least in part, by the relative invulnerability of the corresponding targets.^33^ The identification of CoaBC as a bactericidal target in the CoA biosynthesis pathway in Mtb^33^ therefore prompted us to focus our efforts on this bifunctional enzyme. This led to the first reported crystal structure of the bifunctional CoaBC from *M. smegmatis* and identification of two distinct chemical scaffolds that potently inhibit the CoaB domain of Mtb CoaBC.^41^ Although neither inhibitor showed significant whole-cell activity against Mtb, these promising biochemical and structural data reinforced the potential of CoaBC as a target and encouraged us to seek alternative inhibitory scaffolds.

A high-throughput screen for inhibitors of the CoaB activity of Mtb CoaBC yielded a primary screening hit, **1a**, with activity against the enzyme in the low micromolar range (IC_50_ = 9.9 μM); however, this compound was inactive against replicating Mtb (MIC > 125 μM). Limited exploration around compound **1a** led to the identification of an analogue, **1f**, which showed similar activity against the enzyme (IC_50_ = 15.6 μM) but displayed modest whole-cell inhibitory activity against Mtb (MIC = 25.9 μM). Importantly, the observed ATc-dependent hypersensitization of a *coaBC* hypomorph^33^ to this compound suggested that it engages CoaBC in whole Mtb cells. This conclusion is supported by two further lines of evidence: first, supplementation of the culture medium with PantS, which specifically enables CoaBC bypass, rescued Mtb from **1f** toxicity, thus establishing a direct link between CoaBC engagement and growth inhibition. Second, **1f** elicited a metabolomic profile with distinctive features consistent with CoA pathway disruption and reminiscent of that observed upon transcriptional silencing of *coaBC*, as evidenced by accumulation of dephospho-CoA and dose-dependent depletion of the thioesters, propionyl-, succinyl-, and malonyl-CoA, which are involved in multiple cellular processes. However, unlike transcriptional silencing of *coaBC* for 3 days, exposure of Mtb to **1f** for 24 h had no discernible impact on the level of CoA. While this may reflect the longevity of this cofactor,^54^ it is worth noting that genetic inhibition of a target (via its depletion within the cell) is an imperfect surrogate for chemical inhibition of that target,^44,47,58^ which complicates direct comparison of the metabolic consequences of transcriptional silencing vs. drug treatment.

As noted above, the substantial accumulation of dephospho-CoA and lack of accumulation of PPA following exposure of Mtb to compounds **1f** and **2b** as well as in response to *coaBC* silencing were not readily predicted, consistent with our assertion that the impact of CoaBC inhibition is more complex than alteration of its specific substrate and product levels alone.^33^ PanK, which catalyzes the first committed step in the conversion of Pan to CoA, is considered the rate-limiting enzyme in the CoA biosynthesis pathway^59^ and is regulated by feedback inhibition by CoA and its thioesters.^60^ However, Mtb CoaD is also regulated by feedback inhibition by CoA, as well as by dephospho-CoA, the product of its own reaction,^27,61−63^ and we recently reported that CoA and several acyl-CoAs are additionally capable of inhibiting CoaB activity in Mtb.^41^ Together, these observations suggest that the regulatory mechanisms mediating CoA biosynthesis in Mtb are complex and incompletely understood. As such, it is interesting to speculate that the depletion of ATP observed following exposure of Mtb to compounds **1f** and **2b** (Figure 4) may contribute to the observed accumulation of dephospho-CoA by reducing the catalytic efficiency of the ATP-dependent Mtb CoaE,^64^ which catalyzes the final step in the pathway by conversion of dephospho-CoA to CoA. Additionally, Mtb CoaE is competitively inhibited by CTP, for which it has an affinity similar to that of its substrate, dephospho-CoA.^64^ Under physiological conditions, CoaE is predominantly found in an inactive trimeric form, which is converted to an active monomeric form in the presence of dephospho-CoA.^65^ However, due to overlap of the dephospho-CoA and CTP binding sites, binding of CTP to CoaE completely abolishes its enzymatic activity by preventing conversion to the active form despite the presence of dephospho-CoA, with 100 μM CTP resulting in a greater than 60% decrease in CoaE activity.^65^ Given that **1f** displays noncompetitive (but very close to competitive) inhibition for CTP (*K*_i_ = 10.1 ± 1.4 μM) and that Mtb CoaB exhibits an apparent *K*_m_ of 27.4 ± 2.6 μM for CTP, the inhibitory activity of **1f** may cause a transient increase in intracellular CTP pools, thus limiting the catalytic efficiency of CoaE and contributing to the observed accumulation of dephospho-CoA. This hypothesis is further supported by the observation that Mtb CoaBC can bind PPA in the absence of CTP, triggering the formation of a dead-end complex in which PPA and **1f** are bound to the free enzyme, thereby preventing the binding of CTP. Notably, this mode of binding, with both **1f** and **2b** showing uncompetitive inhibition for PPA, could also account for the lack of accumulation of this CoaBC substrate observed following exposure of Mtb to both of these compounds for 24 h. Importantly, however, despite the induction of these unpredictable metabolomic alterations, the comparable signatures observed following exposure of Mtb to **1f** or **2b** and upon transcriptional silencing of *coaBC* provide further evidence in support of the target selectivity of these compounds.

The lack of correlation between CoaBC inhibition and whole-cell activity observed for the other compounds investigated in this study provides yet another example of the profound challenges of target-based TB drug discovery.^3^ In this regard, the chemical validation of CoaBC resulting from the identification of **1f** as a compound that can penetrate Mtb, evade metabolism, and engage its cellular target, thereby inhibiting Mtb growth, represents a significant advance and provides a platform for further drug discovery efforts. Although **1f** lacks selectivity with respect to the human enzyme, ongoing efforts to exploit additional chemical properties of these molecules are underway, with the aim of identifying compounds that not only show selectivity for Mtb CoaB but also demonstrate more potent inhibition of biochemical and phenotypic activity. The finding that **1f** uncompetitively inhibits the mycobacterial CoaB, binding preferentially in the presence of PPA, presents a mode of inhibition distinct from those previously described^41^ and highlights the versatility of Mtb CoaB as a target that is potentially amenable to chemical inhibition via multiple approaches and by a variety of diverse chemical scaffolds.

## Methods

### Compound Synthesis

Solvents and reagents were purchased from commercial suppliers and used without further purification. Dry solvents were purchased in sure sealed bottles stored over molecular sieves. Reactions using microwave irradiation were carried out in a Biotage Initiator microwave. Normal phase thin layer chromatography (TLC) was performed using precoated silica plates (Kieselgel 60 F254, BDH) with visualization via UV light (UV254/365 nm) or ninhydrin solution. Flash chromatography was performed using Combiflash Companion Rf (Teledyne ISCO) and prepacked silica gel columns (Teledyne ISCO). Mass-directed preparative high performance liquid chromatography (HPLC) separations were performed using an Agilent 1260 Infinity system equipped with diode array detector (DAD) and mass detector, using Waters Sunfire C18 OBD Prep Column, 100 Å, 5 μm, 19 mm × 100 mm with SunFire C18 Prep Guard Cartridge, 100 Å, 10 μm, 19 mm × 10 mm; using water (solvent A) and methanol (solvent B). ^1^H NMR spectra were recorded on a Bruker Avance DPX 500 spectrometer (^1^H at 500.1 MHz, ^13^C at 125 MHz, ^19^F at 470.5 MHz), or a Bruker Avance DPX 400 (^1^H at 400 MHz). Chemical shifts (δ) are expressed in ppm, using the residual solvent as the internal reference in all cases. Signal splitting patterns are described as singlet (s), doublet (d), triplet (t), quartet (q), multiplet (m), broad (br), or a combination thereof. Coupling constants (*J*) are quoted to the nearest 0.1 Hz.

High resolution mass spectrometry (HRMS) was carried out using a Bruker Daltonics MicrOTOF mass spectrometer run in positive mode. LC-MS analysis and chromatographic separation were conducted using (i) Bruker Daltonics MicrOTOF mass spectrometer or an Agilent Technologies 1200 series HPLC connected to an Agilent Technologies 6130 quadrupole LC/MS, where both instruments were connected to an Agilent diode array detector, (ii) Bruker MicrOTOF II focus ESI mass spectrometer connected in parallel to a Thermo Dionex Ultimate 3000 RSLC system with diode array detector, (iii) Advion Expression mass spectrometer connected to a Thermo Dionex Ultimate 3000 HPLC with diode array detector, using either a Waters XBridge column (50 mm × 2.1 mm, 3.5 μm particle size) or a Waters X-select column (30 mm × 2.1 mm, 2.5 μm particle size) with a gradient of 5–90% acetonitrile/water + 0.1% formic acid, or (iv) Shimadzu LCMS-2020 single quadrupole liquid chromatograph mass spectrometer (LC/MS) system equipped with a Hypersil gold column 50 mm × 2.1 mm 1.9 μm pore size. All assay compounds had a measured purity of ≥95% as determined using these analytical LC-MS systems.

#### Compound **1a**, 4-Phenylsulfanyl-5-methyl-2-phenyl-pyrazol-3-ol

To a stirred solution of 5-methyl-2-phenyl-pyrazol-3-ol (50 mg, 0.29 mmol) in acetonitrile (3 mL) were added benzenethiol (47.4 mg, 0.43 mmol) and sodium hydroxide (13.8 mg, 0.34 mmol), and the reaction was heated at 80 °C for 18 h. The reaction mixture was concentrated *in vacuo* to give a crude residue, which was purified by column chromatography (SiO_2_, 0–100% ethyl acetate:hexane) to give the title compound (58 mg, 0.20 mmol, 68.0% yield) as a yellow solid.

^1^H NMR (500 MHz, DMSO-*d*_6_) δ 12.19 (s, 1H), 7.75 (d, *J* = 7.6 Hz, 2H), 7.48 (dd, *J* = 7.9, 7.9 Hz, 2H), 7.32–7.26 (m, 3H), 7.15–7.07 (m, 4H), 2.13 (s, 3H). HRMS ES^+^ 283.0895, C_16_H_15_N_2_OS requires 283.0900.

#### Compound **1b**, 4-(4-Methylphenyl)sulfanyl-5-methyl-2-phenyl-pyrazol-3-ol

To a stirred solution of 5-methyl-2-phenyl-pyrazol-3-ol (50 mg, 0.29 mmol) in acetonitrile (3 mL) were added sodium 4-methylbenzenethiolate (42 mg, 0.29 mmol) and sodium hydroxide (13.8 mg, 0.34 mmol), and the reaction was heated at 80 °C for 18 h. The reaction mixture was concentrated *in vacuo* to give a crude residue, which was purified by column chromatography (SiO_2_, 0–100% ethyl acetate/hexane) to give the title compound (41 mg, 0.13 mmol, 46.7% yield) as a yellow solid.

^1^H NMR (500 MHz, DMSO-*d*_6_) δ 12.13 (s, 1H), 7.74 (d, *J* = 7.6 Hz, 2H), 7.48 (dd, *J* = 8.0, 8.0 Hz, 2H), 7.29 (t, *J* = 7.3 Hz, 1H), 7.11 (d, *J* = 8.1 Hz, 2H), 7.00 (d, *J* = 7.8 Hz, 2H), 2.24 (s, 3H), 2.09 (s, 3H). HRMS ES^+^ 297.1043, C_17_H_14_N_2_OS requires 297.1056.

#### Compound **1c**, 4-(4-Chlorophenyl)sulfanyl-5-methyl-2-phenyl-pyrazol-3-ol

To a stirred solution of 5-methyl-2-phenyl-pyrazol-3-ol (55 mg, 0.32 mmol) in acetonitrile (3 mL) were added 4-chlorobenzenethiol (68.5 mg, 0.48 mmol) and sodium hydroxide (15.1 mg, 0.38 mmol), and the reaction was heated at 80 °C for 18 h. The reaction mixture was concentrated *in vacuo* to give a crude residue, which was purified by column chromatography (SiO_2_, 0–100% ethyl acetate/hexane) to give the title compound (25 mg, 0.07 mmol, 23.5% yield) as a yellow solid.

^1^H NMR (500 MHz, DMSO-*d*_6_) δ 12.30 (s, 1H), 7.75 (d, *J* = 7.6 Hz, 2H), 7.50–7.46 (m, 2H), 7.35 (d, *J* = 8.5 Hz, 2H), 7.29 (t, *J* = 7.4 Hz, 1H), 7.10 (d, *J* = 8.5 Hz, 2H), 2.12 (s, 3H). HRMS ES^+^ 317.0488, C_16_H_14_N_2_OSCl requires 317.0509.

#### Compound **1d**, 4-(3,4-Dichlorophenyl)sulfanyl-5-methyl-2-phenyl-pyrazol-3-ol

To a stirred solution of 5-methyl-2-phenyl-pyrazol-3-ol (55 mg, 0.32 mmol) in acetonitrile (3 mL) were added 3,4-dichlorobenzenethiol (77.1 mg, 0.43 mmol) and sodium hydroxide (15.1 mg, 0.38 mmol), and the reaction was heated at 80 °C for 18 h. The reaction mixture was concentrated *in vacuo* to give a crude residue, which was purified by column chromatography (SiO_2_, 0–100% ethyl acetate/hexane) to give the title compound (58 mg, 0.16 mmol, 54.6% yield) as a yellow solid.

^1^H NMR (500 MHz, DMSO-*d*_6_) δ 12.38 (s, 1H), 7.77–7.75 (m, 2H), 7.64 (d, *J* = 2.3 Hz, 1H), 7.51–7.48 (m, 2H), 7.37–7.29 (m, 2H), 6.78 (d, *J* = 7.0 Hz, 1H), 2.13 (s, 3H). HRMS ES^+^ 351.0122, C_16_H_13_N_2_OSCl_2_ requires 351.0120.

#### Compound **1e**, 4-(4-Methoxyphenyl)sulfanyl-5-methyl-2-phenyl-pyrazol-3-ol

To a stirred solution of 5-methyl-2-phenyl-pyrazol-3-ol (55 mg, 0.32 mmol) in acetonitrile (3 mL) were added 4-methoxybenzenethiol (66.4 mg, 0.47 mmol) and sodium hydroxide (15.1 mg, 0.38 mmol), and the reaction was heated at 80 °C for 18 h. The reaction mixture was concentrated *in vacuo* to give a crude residue, which was purified by column chromatography (SiO_2_, 0–100% ethyl acetate/hexane) to give the title compound (25 mg, 0.07 mmol, 24% yield) as a yellow solid.

^1^H NMR (500 MHz, DMSO-*d*_6_) δ 12.10 (m, 1H), 7.73 (d, *J* = 7.6 Hz, 2H), 7.49–7.45 (m, 2H), 7.27 (t, *J* = 7.3 Hz, 1H), 7.09 (d, *J* = 8.3 Hz, 2H), 6.89 (d, *J* = 8.8 Hz, 2H), 3.71 (s, 3H), 2.11 (s, 3H). HRMS ES^+^ 313.0983, C_17_H_17_N_2_O_2_S requires 313.1005.

#### Compound **1f**, 4-(5-Chloro-1,3-benzoxazol-2-yl)sulfanyl-5-methyl-2-phenyl-pyrazol-3-ol

To a stirred solution of 5-methyl-2-phenyl-pyrazol-3-ol (75 mg, 0.43 mmol) in acetonitrile (3 mL) were added 5-chloro-1,3-benzoxazole-2-thiol (119.8 mg, 0.65 mmol) and sodium hydroxide (20.7 mg, 0.52 mmol), and the reaction was heated at 80 °C for 18 h. The reaction mixture was concentrated *in vacuo* to give a crude residue, which was purified by column chromatography (SiO_2_, 0–100% ethyl acetate:hexane) to give the title compound (15 mg, 0.04 mmol, 9.3% yield) as a yellow solid.

^1^H NMR (500 MHz, DMSO-*d*_6_) δ 12.10 (s, 1H), 7.81–7.79 (m, 2H), 7.68–7.65 (m, 1H), 7.53–7.39 (m, 3H), 7.33–7.26 (m, 2H), 2.07 (s, 3H). HRMS ES^+^ 358.0378, C_17_H_13_N_2_O_2_SCl requires 358.0411.

#### Compound **1g**, 2-(4-Chlorophenyl)-4-(4-chlorophenyl)sulfanyl-5-methyl-pyrazol-3-ol

##### Step 1, 2-(4-Chlorophenyl)-5-methyl-pyrazol-3-ol

To a stirred solution of 4-chlorophenylhydrazine hydrochloride (275 mg, 1.54 mmol) in acetic acid (3 mL) was added ethyl-3-oxobutanoate (200 mg, 1.54 mmol), and the reaction was heated at reflux for 3 h. The solvent was removed *in vacuo* to give a crude residue, which was purified by column chromatography (SiO_2_, 0–100% ethyl acetate/hexane) to give the title compound (115 mg, 0.5 mmol, 32.2% yield), which was used without further purification. LRMS (ESI) *m*/*z* [MH^+^] = 209.1.

##### Step 2, 2-(4-Chlorophenyl)-4-(4-chlorophenyl)sulfanyl-5-methyl-pyrazol-3-ol

To a stirred solution of 2-(4-chlorophenyl)-5-methyl-pyrazol-3-ol (93 mg, 0.43 mmol) in acetonitrile (3 mL) were added 4-chlorothiophenol (96.7 mg, 0.67 mmol) and sodium hydroxide (21.4 mg, 0.54 mmol), and the reaction was heated at 80 °C for 18 h. The reaction mixture was concentrated *in vacuo* to give a crude residue, which was purified by column chromatography (SiO_2_, 0–100% ethyl acetate/hexane) to give the title compound (70.3 mg, 0.19 mmol, 42.7% yield) as a yellow solid.

^1^H NMR (500 MHz, DMSO-*d*_6_) δ 12.43 (s, 1H), 7.82–7.78 (m, 2H), 7.56–7.53 (m, 2H), 7.36–7.33 (m, 2H), 7.12–7.08 (m, 2H), 2.13 (s, 3H). HRMS ES^+^ 351.0120, C_16_H_13_N_2_O_2_SCl_2_ requires 351.0121.

#### Compound **1h**, 2-(3-Chlorophenyl)-4-(4-chlorophenyl)sulfanyl-5-methyl-pyrazol-3-ol

To a stirred solution of ethyl 2-chloro-3-oxobutanoate (45.9 mg, 0.28 mmol) in DMF (1 mL) were added 4-chlorobenzenethiol (40.3 mg, 0.28 mmol) and DIPEA (58.3 μL, 0.34 mmol); the reaction was stirred at room temperature for 1 h and then heated at 100 °C for 9 h. The solvent was removed *in vacuo* to give a crude residue, which was dissolved in acetic acid (0.7 mL). To this (3-chlorophenyl)hydrazine hydrochloride (50 mg, 0.28 mmol) and sodium acetate (25 mg, 0.31 mmol) were added, and the reaction mixture stirred at room temperature for 1 h followed by heating at 100 °C for 12 h.

The solvent was removed *in vacuo* to give a crude residue, which was dissolved in DMSO and filtered, and the filtrate was subjected to HPLC purification to give the title compound (26.7 mg, 0.076 mmol, 27.2% yield) as a pink solid.

^1^H NMR (400 MHz, DMSO-*d*_6_) δ 12.54 (s, 1H), 7.85 (s, 1H), 7.79–7.76 (m, 1H), 7.51 (t, *J* = 8.2 Hz, 1H), 7.36–7.32 (m, 3H), 7.13–7.09 (m, 2H), 2.12 (s, 3H). LRMS (ESI) *m*/*z* [MH^+^] = 351.0.

#### Compound **1i**, 2-(4-Chlorophenyl)-4-(4-chlorophenyl)sulfanyl-5-methyl-pyrazol-3-ol

##### Step 1, 2-(5-Chloro-2-pyridyl)-5-methyl-pyrazol-3-ol

To a stirred solution of (5-chloro-2-pyridyl)hydrazine (220.6 mg, 1.54 mmol) in acetic acid (3 mL) was added ethyl-3-oxobutanoate (200 mg, 1.54 mmol), and the reaction was heated at reflux for 3 h. The solvent was removed *in vacuo* to give a crude residue, which was purified by column chromatography (SiO_2_, 0–100% ethyl acetate/hexane) to give the title compound (242 mg, 1.1 mmol, 71.3% yield) as an off-white solid.

^1^H NMR (500 MHz, DMSO-*d*_6_) δ 12.03 (s, 1H), 8.46 (d, *J* = 2.6 Hz, 1H), 8.40 (s, 1H), 8.02 (dd, *J* = 2.6, 9.0 Hz, 1H), 5.16 (s, 1H), 2.17 (s, 3H). LRMS (ESI) *m*/*z* [MH^+^] = 210.1

##### Step 2, 2-(4-Chlorophenyl)-4-(4-chlorophenyl)sulfanyl-5-methyl-pyrazol-3-ol

To a stirred solution of 2-(5-chloro-2-pyridyl)-5-methyl-pyrazol-3-ol (96 mg, 0.46 mmol) in acetonitrile (3 mL) were added 4-chlorothiophenol (99.3 mg, 0.69 mmol) and sodium hydroxide (22 mg, 0.55 mmol), and the reaction was heated at 80 °C for 18 h. The reaction mixture was concentrated *in vacuo* to give a crude residue, which was purified by column chromatography (SiO_2_, 0–100% ethyl acetate/hexane) to give the title compound (96 mg, 0.26 mmol, 55.9% yield) as a yellow solid.

^1^H NMR (500 MHz, DMSO-*d*_6_) δ 12.43 (s, 1H), 7.82–7.78 (m, 2H), 7.56–7.53 (m, 2H), 7.36–7.33 (m, 2H), 7.12–7.08 (m, 2H), 2.13 (s, 3H). LRMS (ESI) *m*/*z* [MH^+^] = 352.1.

### High-Throughput Screening of Small Molecule Libraries

A library of 215 000 small molecules from the DDU compound collection was screened to identify potential inhibitors of Mtb CoaB at 30 μM using an adaptation of the BIOMOL Green (Enzo Life Sciences) pyrophosphate quantification assay, as previously described.^41^ Briefly, decreased production of phosphate following addition of a pyrophosphatase was indicative of reduced pyrophosphate production by CoaB and, hence, inhibition.

### Counter-screening of Primary Screen Hits

Counter-screens carried out to exclude false positive compounds were performed in 50 μL reactions containing 0.5 U/mL pyrophosphatase and 2 μM pyrophosphate in 100 mM Tris-HCl, pH 7.6, buffer containing 1 mM MgCl_2_ and 1 mM TCEP for 2 h at room temperature in 384-well microplates (Greiner Bio-One). Control reactions were performed in the absence of the pyrophosphatase. The reactions were terminated by addition of 50 μL BIOMOL Green (Enzo Life Sciences), and product formation was measured by absorbance (650 nm) using a PHERAstar microplate reader (BMG Labtech) following incubation for 20 min. Liquid dispensing was performed using a Thermo Scientific Matrix Wellmate, and data were processed and analyzed using ActivityBase (IDBS).

### Pharmacological Profiling of Compounds

Compounds were profiled for HepG2 cytotoxicity, mouse liver microsomal clearance, and aqueous solubility, as previously described.^66^

### Bacterial Strains and Growth Conditions

The *coaBC* Tet-OFF hypomorph used in this study was constructed as previously described,^33^ and derived from the virulent, PDIM-producing parental strain, Mtb H37RvMA.^42^ All strains were routinely grown in Difco Middlebrook 7H9 broth (BD) supplemented with Middlebrook albumin-dextrose-catalase (ADC) enrichment (BD), 0.2% glycerol (Sigma-Aldrich), and 0.05% Tween-80, unless otherwise indicated. Hygromycin and kanamycin were used at concentrations of 50 and 25 μg/mL, and pantethine (Sigma-Aldrich) was included at 2.5 mg/mL where required. In order to repress the expression of target genes in cells expressing revTetR, cultures were routinely grown in the absence of supplementation to OD_600_ ≈ 0.2 prior to dilution in Middlebrook 7H9 broth containing the ATc inducer at concentrations up to 200 ng/mL in order to transcriptionally silence *coaBC*. To avoid inactivation of the inducer, all cultures containing ATc were incubated in the dark, and exposure of the cultures to light was minimized.

### MICs and Checkerboard Assays for Assessing Target Selectivity

The minimum inhibitory concentrations (MICs) of the compounds were determined using the microbroth dilution assay and growth inhibition was quantitatively determined using Alamar Blue as a measure of fluorescence output, as previously described.^46^ Briefly, 2-fold serial dilutions of each compound were inoculated with a suspension of Mtb H37RvMA^42^ or the *coaBC* hypomorph^33^ at cell densities of ∼10^5^ CFU/mL (OD_600_ = 0.6) and ∼10^4^ CFU/mL (OD_600_ = 0.2), respectively, to a final volume of 100 μL in a U-bottom 96-well microtiter plate and incubated at 37 °C for 10 days. A volume of 10 μL of Alamar Blue solution was then added to each well, and the plates were incubated at 37 °C for a further 24 h. Fluorescence as an indication of growth was measured using a SpectraMax i3x multimode microplate reader (Molecular Devices) in bottom-reading mode with excitation at 544 nm and emission at 590 nm. All data are representative of independent triplicates.

### Protein Purification and Enzymatic Assays

*E. coli* BL21(DE3) containing a pET28aSUMO-CoaBC construct or a human CoaB construct with a cleavable N-terminal 6×His tag was grown to mid-exponential growth phase (OD_600_ = 0.6–0.8) in 2×YT media supplemented with kanamycin (30 μg/mL) at 37 °C. Overexpression of the enzymes was induced by the addition of 0.5 mM IPTG and incubation at 18 °C for 18–20 h. Both enzymes were purified, and enzymatic assays were performed using the EnzChek pyrophosphate assay kit (E-6645) (Life Technologies) except in the case of l-cysteine competition, where a CMP quantification assay was used, as previously described.^41^ Briefly, the IC_50_ for compound **1f** was determined in 100 mM Tris, pH 7.5, 1 mM TCEP, 2% DMSO, 1 mM MgCl_2_, 200 μM MSEG, 0.03 U/mL inorganic pyrophosphatase, 1 U/mL purine nucleoside phosphorylase, with 32 nM Mtb CoaBC, 125 μM CTP, 125 μM PPA, 500 μM l-cysteine, and variable concentrations of **1f** (2–256 μM). In the assays with human CoaB, an enzyme concentration of 1 μM was used. The inhibition of the coupling enzymes by **1f** was assessed, and the compound was inactive against these enzymes at the tested range of concentrations. Kinetic parameters and competition assays for CTP and PPA were determined under the same conditions but with variable concentrations of CTP (31.25 μM, 62.5 μM, 125 μM, 250 μM, and 500 μM) with 125 μM PPA and 500 μM l-cysteine or variable concentrations of PPA (31.25 μM, 62.5 μM, 125 μM, 250 μM, and 500 μM) with 125 μM CTP and 500 μM l-cysteine.

Kinetic parameters and competition assays for l-cysteine were determined in 1 mM MgCl_2_, 100 mM Tris, pH 7.5, 1 mM TCEP, 2% DMSO, 32 nM Mtb CoaBC, 125 μM CTP, 125 μM PPA, and variable concentrations of l-cysteine (31.25 μM, 62.5 μM, 93.75 μM, 125 μM, and 250 μM). All reactions were carried out in at least triplicate. IC_50_, Michaelis–Menten, and inhibition constants were calculated using GraphPad Prism.

### Differential Scanning Fluorimetry

Differential scanning fluorimetry (DSF) was used to assess inhibitor binding to the CoaB domain of CoaBC. The assay was performed in a 96 well plate format using a CFX Connect (Bio-Rad). Each well contained a solution of 5 μM *M. tuberculosis* CoaBC, 25 mM Tris, pH 8.0, 150 mM NaCl, 1 mM MgCl_2_, 2.5% DMSO, 5× Sypro Orange. Substrates or products were added individually to each well at a final concentration of 1 mM in the presence and absence of 2.5 mM **1f**. Combinations of substrates and products (CMP and l-cysteine; CTP and l-cysteine; CMP and PPA; CTP and PPA; CMP, PPA, and l-cysteine; PPA and l-cysteine) each at a final concentration of 1 mM were also tested in the presence or absence of 2.5 mM **1f**. Unfolding of CoaBC in the absence of any substrate or product was measured in parallel. All data are representative of independent triplicates.

### Metabolite Extraction and Metabolomic Profiling

Mtb-laden filters used for metabolomic analyses were generated as previously described^53^ and incubated at 37 °C for 4 days to expand biomass. Mtb-laden filters were then transferred onto a fresh 7H10 agar plate with or without **1f** and **2b** at 5 and 50 μM for a further 24 h. Metabolite extraction and LC-MS-based metabolomic profiling were carried out as previously described.^33^ All data obtained by metabolomics were the average of independent triplicates.

### *Mycobacterium tuberculosis* Compound Uptake

Compound uptake was determined using a filter culture system, as previously described.^41,53^ Briefly, Mtb was grown on nylon Durapore 0.22 μm membrane filters placed on top of Middlebrook 7H11 agar plates supplemented with 0.2% glycerol and 10% OADC for 1 week at 37 °C to expand the biomass. The membranes were then placed atop a reservoir containing Middlebrook 7H9 broth with or without compound, such that the underside of the bacteria-laden filters was in direct contact with the media. Following 24 h of incubation at 37 °C, which we previously determined to be pre-lethal for most frontline drugs,^11^ the filters were plunged into 40:40:20 methanol:acetonitrile:water precooled to −20 °C, and cells were lysed using a bead beater. Cell lysates were mixed with equal volumes of 50% acetonitrile and 0.2% formic acid, and drug accumulation was measured using mass spectrometry in positive and negative ion mode, as previously described.^53^ Relative drug levels were quantified by comparison with standard curves generated from bacterial lysates spiked with compound, using the method of standard addition.^67^ Internal standards were routinely included with each sample run, and data were additionally normalized to sample protein biomass.

## Supporting Information Available

Effect of exogenous supplementation with pantothenate on the growth of CoaBC- and PanC-deficient Mtb, lack of effect ATc on the susceptibility of Mtb H37RvMA to compound **1f** when assessed at low cell density, Mtb CoaBC saturation curve fit for Michaelis–Menten model, DSF melting profile of CoaBC, movement of a flexible loop covering PPA binding site, impact of exposure of Mtb H37Rv to **1f** and **2b** on acyl-CoA species and CoA pathway metabolites, total intracellular accumulation of compound **1f** and **2b**, and standard curves showing comparable ionization efficiencies of compounds **1f** and **2b** spiked into bacterial extracts (PDF)

## Abbreviations

ATc: anhydrotetracycline
CMP: cytidine-5′-monophosphate
CoaB: 4′-phosphopantothenoyl-l-cysteine synthetase (PPCS)
CoaC: 4′-phosphopantothenoyl-cysteine decarboxylase (PPCDC)
CTP: cytidine-5′-triphosphate
DSF: differential scanning fluorimetry
EthA: monooxygenase
Mtb: *Mycobacterium tuberculosis*
Pan: pantothenate (vitamin B_5_)
PanC: pantothenate synthetase
PanD: aspartate decarboxylase
PanK (CoaA): type I pantothenate kinase
PantS: pantethine
PantSH: pantetheine
PPA: 4′-phosphopantothenate
P-PantSH: 4′-phosphopantetheine
PptT: phosphopantetheinyl transferase
TCEP: tris(2-carboxyethyl)phosphine
PDIM: phthiocerol dimycocerosate
OADC: oleic-albumin-dextrose-catalase

## References

[ref1] Global Tuberculosis Report, WHO. 2020.

[ref2] Zumla A.; Nahid P.; Cole S. T. (2013) Advances in the development of new tuberculosis drugs and treatment regimens. Nat. Rev. Drug Discovery 12 (5), 388–404. 10.1038/nrd4001. 23629506

[ref3] Evans J. C.; Mizrahi V. (2018) Priming the tuberculosis drug pipeline: new antimycobacterial targets and agents. Curr. Opin. Microbiol. 45, 39–46. 10.1016/j.mib.2018.02.006. 29482115

[ref4] J Libardo M D.; Boshoff H. I.; Barry C. E (2018) The present state of the tuberculosis drug development pipeline. Curr. Opin. Pharmacol. 42, 81–94. 10.1016/j.coph.2018.08.001. 30144650 PMC6204086

[ref5] Campanico A.; Moreira R.; Lopes F. (2018) Drug discovery in tuberculosis. New drug targets and antimycobacterial agents. Eur. J. Med. Chem. 150, 525–545. 10.1016/j.ejmech.2018.03.020. 29549838

[ref6] Conradie F.; Everitt D.; Crook A. M. (2020) Treatment of Highly Drug-Resistant Pulmonary Tuberculosis. Reply. N Engl J. Med. 382 (24), 2377 10.1056/NEJMc2009939. 32521143

[ref7] Tiberi S.; Munoz-Torrico M.; Duarte R.; Dalcolmo M.; D’Ambrosio L.; Migliori G. B. (2018) New drugs and perspectives for new anti-tuberculosis regimens. Pulmonology 24 (2), 86–98. 10.1016/j.rppnen.2017.10.009. 29487031

[ref8] Wellington S.; Hung D. T. (2018) The Expanding Diversity of Mycobacterium tuberculosis Drug Targets. ACS Infect. Dis. 4 (5), 696–714. 10.1021/acsinfecdis.7b00255. 29412643

[ref9] Andries K.; Verhasselt P.; Guillemont J.; Gohlmann H. W.; Neefs J. M.; Winkler H.; Van Gestel J.; Timmerman P.; Zhu M.; Lee E.; Williams P.; de Chaffoy D.; Huitric E.; Hoffner S.; Cambau E.; Truffot-Pernot C.; Lounis N.; Jarlier V. (2005) A diarylquinoline drug active on the ATP synthase of Mycobacterium tuberculosis. Science 307 (5707), 223–7. 10.1126/science.1106753. 15591164

[ref10] Matsumoto M.; Hashizume H.; Tomishige T.; Kawasaki M.; Tsubouchi H.; Sasaki H.; Shimokawa Y.; Komatsu M. (2006) OPC-67683, a nitro-dihydro-imidazooxazole derivative with promising action against tuberculosis in vitro and in mice. PLoS Med. 3 (11), e466 10.1371/journal.pmed.0030466. 17132069 PMC1664607

[ref11] Nandakumar M.; Nathan C.; Rhee K. Y. (2014) Isocitrate lyase mediates broad antibiotic tolerance in Mycobacterium tuberculosis. Nat. Commun. 5, 4306 10.1038/ncomms5306. 24978671

[ref12] Pethe K.; Bifani P.; Jang J.; Kang S.; Park S.; Ahn S.; Jiricek J.; Jung J.; Jeon H. K.; Cechetto J.; Christophe T.; Lee H.; Kempf M.; Jackson M.; Lenaerts A. J.; Pham H.; Jones V.; Seo M. J.; Kim Y. M.; Seo M.; Seo J. J.; Park D.; Ko Y.; Choi I.; Kim R.; Kim S. Y.; Lim S.; Yim S. A.; Nam J.; Kang H.; Kwon H.; Oh C. T.; Cho Y.; Jang Y.; Kim J.; Chua A.; Tan B. H.; Nanjundappa M. B.; Rao S. P.; Barnes W. S.; Wintjens R.; Walker J. R.; Alonso S.; Lee S.; Kim J.; Oh S.; Oh T.; Nehrbass U.; Han S. J.; No Z.; Lee J.; Brodin P.; Cho S. N.; Nam K.; Kim J. (2013) Discovery of Q203, a potent clinical candidate for the treatment of tuberculosis. Nat. Med. 19 (9), 1157–60. 10.1038/nm.3262. 23913123

[ref13] Stover C. K.; Warrener P.; VanDevanter D. R.; Sherman D. R.; Arain T. M.; Langhorne M. H.; Anderson S. W.; Towell J. A.; Yuan Y.; McMurray D. N.; Kreiswirth B. N.; Barry C. E.; Baker W. R. (2000) A small-molecule nitroimidazopyran drug candidate for the treatment of tuberculosis. Nature 405 (6789), 962–6. 10.1038/35016103. 10879539

[ref14] Wellington S.; Nag P. P.; Michalska K.; Johnston S. E.; Jedrzejczak R. P.; Kaushik V. K.; Clatworthy A. E.; Siddiqi N.; McCarren P.; Bajrami B.; Maltseva N. I.; Combs S.; Fisher S. L.; Joachimiak A.; Schreiber S. L.; Hung D. T. (2017) A small-molecule allosteric inhibitor of Mycobacterium tuberculosis tryptophan synthase. Nat. Chem. Biol. 13 (9), 943–950. 10.1038/nchembio.2420. 28671682 PMC6886523

[ref15] Ambady A.; Awasthy D.; Yadav R.; Basuthkar S.; Seshadri K.; Sharma U. (2012) Evaluation of CoA biosynthesis proteins of Mycobacterium tuberculosis as potential drug targets. Tuberculosis (Oxford, U. K.) 92 (6), 521–8. 10.1016/j.tube.2012.08.001. 22954585

[ref16] Cheng C. S.; Jia K. F.; Chen T.; Chang S. Y.; Lin M. S.; Yin H. S. (2013) Experimentally validated novel inhibitors of Helicobacter pylori phosphopantetheine adenylyltransferase discovered by virtual high-throughput screening. PLoS One 8 (9), e74271 10.1371/journal.pone.0074271. 24040220 PMC3764209

[ref17] Li B.; Tempel W.; Smil D.; Bolshan Y.; Schapira M.; Park H. W. (2013) Crystal structures of Klebsiella pneumoniae pantothenate kinase in complex with N-substituted pantothenamides. Proteins: Struct., Funct., Genet. 81 (8), 1466–72. 10.1002/prot.24290. 23553820

[ref18] Spry C.; Kirk K.; Saliba K. J. (2008) Coenzyme A biosynthesis: an antimicrobial drug target. FEMS Microbiol Rev. 32 (1), 56–106. 10.1111/j.1574-6976.2007.00093.x. 18173393

[ref19] van der Westhuyzen R.; Hammons J. C.; Meier J. L.; Dahesh S.; Moolman W. J.; Pelly S. C.; Nizet V.; Burkart M. D.; Strauss E. (2012) The antibiotic CJ-15,801 is an antimetabolite that hijacks and then inhibits CoA biosynthesis. Chem. Biol. 19 (5), 559–71. 10.1016/j.chembiol.2012.03.013. 22633408 PMC3361698

[ref20] Zhao L.; Allanson N. M.; Thomson S. P.; Maclean J. K.; Barker J. J.; Primrose W. U.; Tyler P. D.; Lewendon A. (2003) Inhibitors of phosphopantetheine adenylyltransferase. Eur. J. Med. Chem. 38 (4), 345–9. 10.1016/S0223-5234(03)00047-3. 12750020

[ref21] Butman H. S.; Kotze T. J.; Dowd C. S.; Strauss E. (2020) Vitamin in the Crosshairs: Targeting Pantothenate and Coenzyme A Biosynthesis for New Antituberculosis Agents. Front. Cell. Infect. Microbiol. 10, 721 10.3389/fcimb.2020.605662. PMC7770189 33384970

[ref22] Genschel U. (2004) Coenzyme A biosynthesis: reconstruction of the pathway in archaea and an evolutionary scenario based on comparative genomics. Mol. Biol. Evol. 21 (7), 1242–51. 10.1093/molbev/msh119. 15014152

[ref23] Begley T. P.; Kinsland C.; Strauss E. (2001) The biosynthesis of coenzyme A in bacteria. Vitam. Horm. 61, 157–71. 10.1016/S0083-6729(01)61005-7. 11153265

[ref24] Ballinger E.; Mosior J.; Hartman T.; Burns-Huang K.; Gold B.; Morris R.; Goullieux L.; Blanc I.; Vaubourgeix J.; Lagrange S.; Fraisse L.; Sans S.; Couturier C.; Bacque E.; Rhee K.; Scarry S. M.; Aube J.; Yang G.; Ouerfelli O.; Schnappinger D.; Ioerger T. R.; Engelhart C. A.; McConnell J. A.; McAulay K.; Fay A.; Roubert C.; Sacchettini J.; Nathan C. (2019) Opposing reactions in coenzyme A metabolism sensitize Mycobacterium tuberculosis to enzyme inhibition. Science 363, eaau8959 10.1126/science.aau8959. 30705156 PMC6613350

[ref25] Lee W.; VanderVen B. C.; Fahey R. J.; Russell D. G. (2013) Intracellular Mycobacterium tuberculosis exploits host-derived fatty acids to limit metabolic stress. J. Biol. Chem. 288 (10), 6788–800. 10.1074/jbc.M112.445056. 23306194 PMC3591590

[ref26] Ciulli A.; Scott D. E.; Ando M.; Reyes F.; Saldanha S. A.; Tuck K. L.; Chirgadze D. Y.; Blundell T. L.; Abell C. (2008) Inhibition of Mycobacterium tuberculosis pantothenate synthetase by analogues of the reaction intermediate. ChemBioChem 9 (16), 2606–11. 10.1002/cbic.200800437. 18821554 PMC4441726

[ref27] Timofeev V.; Smirnova E.; Chupova L.; Esipov R.; Kuranova I. (2012) X-ray study of the conformational changes in the molecule of phosphopantetheine adenylyltransferase from Mycobacterium tuberculosis during the catalyzed reaction. Acta Crystallogr., Sect. D: Biol. Crystallogr. 68, 1660–70. 10.1107/S0907444912040206. 23151631

[ref28] Venkatraman J.; Bhat J.; Solapure S. M.; Sandesh J.; Sarkar D.; Aishwarya S.; Mukherjee K.; Datta S.; Malolanarasimhan K.; Bandodkar B.; Das K. S. (2012) Screening, identification, and characterization of mechanistically diverse inhibitors of the Mycobacterium tuberculosis enzyme, pantothenate kinase (CoaA). J. Biomol. Screening 17 (3), 293–302. 10.1177/1087057111423069. 22086722

[ref29] Xu Z.; Yin W.; Martinelli L. K.; Evans J.; Chen J.; Yu Y.; Wilson D. J.; Mizrahi V.; Qiao C.; Aldrich C. C. (2014) Reaction intermediate analogues as bisubstrate inhibitors of pantothenate synthetase. Bioorg. Med. Chem. 22 (5), 1726–35. 10.1016/j.bmc.2014.01.017. 24507827 PMC4667779

[ref30] Bjorkelid C.; Bergfors T.; Raichurkar A. K.; Mukherjee K.; Malolanarasimhan K.; Bandodkar B.; Jones T. A. (2013) Structural and biochemical characterization of compounds inhibiting Mycobacterium tuberculosis pantothenate kinase. J. Biol. Chem. 288 (25), 18260–70. 10.1074/jbc.M113.476473. 23661699 PMC3689968

[ref31] Reddy B. K.; Landge S.; Ravishankar S.; Patil V.; Shinde V.; Tantry S.; Kale M.; Raichurkar A.; Menasinakai S.; Mudugal N. V.; Ambady A.; Ghosh A.; Tunduguru R.; Kaur P.; Singh R.; Kumar N.; Bharath S.; Sundaram A.; Bhat J.; Sambandamurthy V. K.; Bjorkelid C.; Jones T. A.; Das K.; Bandodkar B.; Malolanarasimhan K.; Mukherjee K.; Ramachandran V. (2014) Assessment of Mycobacterium tuberculosis pantothenate kinase vulnerability through target knockdown and mechanistically diverse inhibitors. Antimicrob. Agents Chemother. 58 (6), 3312–26. 10.1128/AAC.00140-14. 24687493 PMC4068421

[ref32] Kumar A.; Casey A.; Odingo J.; Kesicki E. A.; Abrahams G.; Vieth M.; Masquelin T.; Mizrahi V.; Hipskind P. A.; Sherman D. R.; Parish T. (2013) A high-throughput screen against pantothenate synthetase (PanC) identifies 3-biphenyl-4-cyanopyrrole-2-carboxylic acids as a new class of inhibitor with activity against Mycobacterium tuberculosis. PLoS One 8 (11), e72786 10.1371/journal.pone.0072786. 24244263 PMC3820577

[ref33] Evans J. C.; Trujillo C.; Wang Z.; Eoh H.; Ehrt S.; Schnappinger D.; Boshoff H. I.; Rhee K. Y.; Barry C. E. 3rd; Mizrahi V. (2016) Validation of CoaBC as a Bactericidal Target in the Coenzyme A Pathway of Mycobacterium tuberculosis. ACS Infect. Dis. 2 (12), 958–968. 10.1021/acsinfecdis.6b00150. 27676316 PMC5153693

[ref34] Strauss E.; Kinsland C.; Ge Y.; McLafferty F. W.; Begley T. P. (2001) Phosphopantothenoylcysteine synthetase from Escherichia coli. Identification and characterization of the last unidentified coenzyme A biosynthetic enzyme in bacteria. J. Biol. Chem. 276 (17), 13513–6. 10.1074/jbc.C100033200. 11278255

[ref35] Kupke T. (2002) Molecular characterization of the 4′-phosphopantothenoylcysteine synthetase domain of bacterial dfp flavoproteins. J. Biol. Chem. 277 (39), 36137–45. 10.1074/jbc.M206188200. 12140293

[ref36] Strauss E.; Begley T. P. (2001) Mechanistic studies on phosphopantothenoylcysteine decarboxylase. J. Am. Chem. Soc. 123 (26), 6449–50. 10.1021/ja016020y. 11427085

[ref37] Moolman W. J.; de Villiers M.; Strauss E. (2014) Recent advances in targeting coenzyme A biosynthesis and utilization for antimicrobial drug development. Biochem. Soc. Trans. 42 (4), 1080–6. 10.1042/BST20140131. 25110006

[ref38] Patrone J. D.; Yao J.; Scott N. E.; Dotson G. D. (2009) Selective inhibitors of bacterial phosphopantothenoylcysteine synthetase. J. Am. Chem. Soc. 131 (45), 16340–1. 10.1021/ja906537f. 19902973 PMC2787235

[ref39] Gopal P.; Sarathy J. P.; Yee M.; Ragunathan P.; Shin J.; Bhushan S.; Zhu J.; Akopian T.; Kandror O.; Lim T. K.; Gengenbacher M.; Lin Q.; Rubin E. J.; Gruber G.; Dick T. (2020) Pyrazinamide triggers degradation of its target aspartate decarboxylase. Nat. Commun. 11, 1661 10.1038/s41467-020-15516-1. 32245967 PMC7125159

[ref40] Gopal P.; Nartey W.; Ragunathan P.; Sarathy J.; Kaya F.; Yee M.; Setzer C.; Manimekalai M. S. S.; Dartois V.; Gruber G.; Dick T. (2017) Pyrazinoic Acid Inhibits Mycobacterial Coenzyme A Biosynthesis by Binding to Aspartate Decarboxylase PanD. ACS Infect. Dis. 3 (11), 807–819. 10.1021/acsinfecdis.7b00079. 28991455 PMC5734868

[ref41] Mendes V. G. S. R.; Evans J. C.; Hess J.; Blaszczyk M.; Spry C.; Bryant O.; Cory-Wright J.; Chan D. S.-H.; Torres P. H. M.; Wang Z.; Nahiyaan N.; O’Neill S.; Damerow S.; Post J.; Bayliss T.; Lynch S. L.; Coyne A. G.; Ray P. C.; Abell C.; Rhee K. Y.; Boshoff H. I. M.; Barry C. E.; Mizrahi V.; Wyatt P. G.; Blundell T. L.; Green S. R. (2021) Inhibiting Mycobacterium tuberculosis CoaBC by targeting an allosteric site. Nat. Commun. 12 (1), 143 10.1038/s41467-020-20224-x. 33420031 PMC7794376

[ref42] Ioerger T. R.; Feng Y.; Ganesula K.; Chen X.; Dobos K. M.; Fortune S.; Jacobs W. R. Jr.; Mizrahi V.; Parish T.; Rubin E.; Sassetti C.; Sacchettini J. C. (2010) Variation among genome sequences of H37Rv strains of Mycobacterium tuberculosis from multiple laboratories. J. Bacteriol. 192 (14), 3645–53. 10.1128/JB.00166-10. 20472797 PMC2897344

[ref43] Abrahams G. L.; Kumar A.; Savvi S.; Hung A. W.; Wen S.; Abell C.; Barry C. E. 3rd; Sherman D. R.; Boshoff H. I.; Mizrahi V. (2012) Pathway-selective sensitization of Mycobacterium tuberculosis for target-based whole-cell screening. Chem. Biol. 19 (7), 844–54. 10.1016/j.chembiol.2012.05.020. 22840772 PMC3421836

[ref44] Evans J. C.; Mizrahi V. (2015) The application of tetracyclineregulated gene expression systems in the validation of novel drug targets in Mycobacterium tuberculosis. Front. Microbiol. 6, 812 10.3389/fmicb.2015.00812. 26300875 PMC4523840

[ref45] Singh V.; Donini S.; Pacitto A.; Sala C.; Hartkoorn R. C.; Dhar N.; Keri G.; Ascher D. B.; Mondesert G.; Vocat A.; Lupien A.; Sommer R.; Vermet H.; Lagrange S.; Buechler J.; Warner D. F.; McKinney J. D.; Pato J.; Cole S. T.; Blundell T. L.; Rizzi M.; Mizrahi V. (2017) The Inosine Monophosphate Dehydrogenase, GuaB2, Is a Vulnerable New Bactericidal Drug Target for Tuberculosis. ACS Infect. Dis. 3 (1), 5–17. 10.1021/acsinfecdis.6b00102. 27726334 PMC5241705

[ref46] Singh V.; Brecik M.; Mukherjee R.; Evans J. C.; Svetlikova Z.; Blasko J.; Surade S.; Blackburn J.; Warner D. F.; Mikusova K.; Mizrahi V. (2015) The complex mechanism of antimycobacterial action of 5-fluorouracil. Chem. Biol. 22 (1), 63–75. 10.1016/j.chembiol.2014.11.006. 25544046

[ref47] Wei J. R.; Krishnamoorthy V.; Murphy K.; Kim J. H.; Schnappinger D.; Alber T.; Sassetti C. M.; Rhee K. Y.; Rubin E. J. (2011) Depletion of antibiotic targets has widely varying effects on growth. Proc. Natl. Acad. Sci. U. S. A. 108 (10), 4176–81. 10.1073/pnas.1018301108. 21368134 PMC3053961

[ref48] El Bakali J. B. M., Evans J. C., Boland J. A., McCarthy W., Dias M. V. B., Coyne A. G., Mizrahi V., Blundell T. L., Abell C., and Spry C. (2020) Fragment Linking Applied to the Discovery of Mycobacterium tuberculosis Phosphopantetheine Adenylyltransferase Inhibitors. bioRxiv, 10.1101/2020.09.04.280388v1.

[ref49] Balibar C. J.; Hollis-Symynkywicz M. F.; Tao J. (2011) Pantethine rescues phosphopantothenoylcysteine synthetase and phosphopantothenoylcysteine decarboxylase deficiency in Escherichia coli but not in Pseudomonas aeruginosa. J. Bacteriol. 193 (13), 3304–12. 10.1128/JB.00334-11. 21551303 PMC3133259

[ref50] Kaskow B. J.; Proffitt J. M.; Blangero J.; Moses E. K.; Abraham L. J. (2012) Diverse biological activities of the vascular non-inflammatory molecules - the Vanin pantetheinases. Biochem. Biophys. Res. Commun. 417 (2), 653–8. 10.1016/j.bbrc.2011.11.099. 22155241 PMC3259148

[ref51] Yao J.; Patrone J. D.; Dotson G. D. (2009) Characterization and kinetics of phosphopantothenoylcysteine synthetase from Enterococcus faecalis. Biochemistry 48 (12), 2799–806. 10.1021/bi802240w. 19182993 PMC2892170

[ref52] Stanitzek S.; Augustin M. A.; Huber R.; Kupke T.; Steinbacher S. (2004) Structural basis of CTP-dependent peptide bond formation in coenzyme A biosynthesis catalyzed by Escherichia coli PPC synthetase. Structure 12 (11), 1977–88. 10.1016/j.str.2004.08.007. 15530362

[ref53] Nandakumar M.; Prosser G. A.; de Carvalho L. P.; Rhee K. (2015) Metabolomics of Mycobacterium tuberculosis. Methods Mol. Biol. 1285, 105–15. 10.1007/978-1-4939-2450-9_6. 25779312

[ref54] Hartl J.; Kiefer P.; Meyer F.; Vorholt J. A. (2017) Longevity of major coenzymes allows minimal de novo synthesis in microorganisms. Nat. Microbiol 2, 17073 10.1038/nmicrobiol.2017.73. 28504670 PMC6241834

[ref55] Awasthi D.; Freundlich J. S. (2017) Antimycobacterial Metabolism: Illuminating Mycobacterium tuberculosis Biology and Drug Discovery. Trends Microbiol. 25 (9), 756–767. 10.1016/j.tim.2017.05.007. 28622844 PMC5564221

[ref56] Mendes V. G. S. R., Evans J. C., Hess J., Blaszczyk M., Spry C., Bryant O., Cory-Wright J., D.S-H C., Torres P. H. M., Wang Z., O’Neill S., Damerow S., Post J., Bayliss T., Lynch S. L., Coyne A. G., Ray P. C., Abell C., Rhee Y. K., Boshoff H. I. M., Barry C. E. III, Mizrahi M., Wyatt P. G., and Blundell T. L (2019) Inhibiting Mycobacterium tuberculosis CoaBC by targeting a new allosteric site. bioRxiv, www.biorxiv.org/content/10.1101/870154v3. 10.1038/s41467-020-20224-x PMC7794376 33420031

[ref57] Chiarelli L. R.; Mori G.; Orena B. S.; Esposito M.; Lane T.; de Jesus Lopes Ribeiro A. L.; Degiacomi G.; Zemanova J.; Szadocka S.; Huszar S.; Palcekova Z.; Manfredi M.; Gosetti F.; Lelievre J.; Ballell L.; Kazakova E.; Makarov V.; Marengo E.; Mikusova K.; Cole S. T.; Riccardi G.; Ekins S.; Pasca M. R. (2018) A multitarget approach to drug discovery inhibiting Mycobacterium tuberculosis PyrG and PanK. Sci. Rep. 8 (1), 3187 10.1038/s41598-018-21614-4. 29453370 PMC5816626

[ref58] Schnappinger D.; Ehrt S. (2014) Regulated Expression Systems for Mycobacteria and Their Applications. Microbiol. Spectrum 10.1128/microbiolspec.MGM2-0018-2013. PMC4254785 25485177

[ref59] Jackowski S.; Rock C. O. (1981) Regulation of coenzyme A biosynthesis. J. Bacteriol. 148 (3), 926–32. 10.1128/JB.148.3.926-932.1981. 6796563 PMC216294

[ref60] Vallari D. S.; Jackowski S.; Rock C. O. (1987) Regulation of pantothenate kinase by coenzyme A and its thioesters. J. Biol. Chem. 262 (6), 2468–71. 10.1016/S0021-9258(18)61527-3. 3029083

[ref61] Wubben T. J.; Mesecar A. D. (2010) Kinetic, thermodynamic, and structural insight into the mechanism of phosphopantetheine adenylyltransferase from Mycobacterium tuberculosis. J. Mol. Biol. 404 (2), 202–19. 10.1016/j.jmb.2010.09.002. 20851704

[ref62] Miller J. R.; Ohren J.; Sarver R. W.; Mueller W. T.; de Dreu P.; Case H.; Thanabal V. (2007) Phosphopantetheine adenylyltransferase from Escherichia coli: investigation of the kinetic mechanism and role in regulation of coenzyme A biosynthesis. J. Bacteriol. 189 (22), 8196–205. 10.1128/JB.00732-07. 17873050 PMC2168673

[ref63] Geerlof A.; Lewendon A.; Shaw W. V. (1999) Purification and characterization of phosphopantetheine adenylyltransferase from Escherichia coli. J. Biol. Chem. 274 (38), 27105–11. 10.1074/jbc.274.38.27105. 10480925

[ref64] Walia G.; Kumar P.; Surolia A. (2009) The role of UPF0157 in the folding of M. tuberculosis dephosphocoenzyme A kinase and the regulation of the latter by CTP. PLoS One 4 (10), e7645 10.1371/journal.pone.0007645. 19876400 PMC2765170

[ref65] Walia G.; Surolia A. (2011) Insights into the regulatory characteristics of the mycobacterial dephosphocoenzyme A kinase: implications for the universal CoA biosynthesis pathway. PLoS One 6 (6), e21390 10.1371/journal.pone.0021390. 21731728 PMC3123319

[ref66] Ray P. C.; Huggett M.; Turner P. A.; Taylor M.; Cleghorn L. A. T.; Early J.; Kumar A.; Bonnett S. A.; Flint L.; Joerss D.; Johnson J.; Korkegian A.; Mullen S.; Moure A. L.; Davis S. H.; Murugesan D.; Mathieson M.; Caldwell N.; Engelhart C. A.; Schnappinger D.; Epemolu O.; Zuccotto F.; Riley J.; Scullion P.; Stojanovski L.; Massoudi L.; Robertson G. T.; Lenaerts A. J.; Freiberg G.; Kempf D. J.; Masquelin T.; Hipskind P. A.; Odingo J.; Read K. D.; Green S. R.; Wyatt P. G.; Parish T. (2021) Spirocycle MmpL3 Inhibitors with Improved hERG and Cytotoxicity Profiles as Inhibitors of Mycobacterium tuberculosis Growth. ACS Omega 6 (3), 2284–2311. 10.1021/acsomega.0c05589. 33521468 PMC7841955

[ref67] Harris D. C. (2003) Quantitative Chemical Analysis, 6th ed., W.H. Freeman, New York.

