# Supplementary material for: Targeting Mycobacterium tuberculosis CoaBC through Chemical Inhibition of 4′-Phosphopantothenoyl-l-cysteine Synthetase (CoaB) Activity
Source: ACS Infect Dis. 2021 May 3;7(6):1666–79. doi: 10.1021/acsinfecdis.0c00904 (PMC8205227; doi:10.1021/acsinfecdis.0c00904)
Supplement: Supplementary file 1 — id0c00904_si_001.pdf [file id0c00904_si_001.pdf]

SUPPORTING INFORMATION (SI)

Targeting *Mycobacterium tuberculosis* CoaBC through chemical inhibition of 4'-phosphopantothenoyl-L-cysteine synthetase (CoaB) activity

Joanna C. Evans[1*¶], Dinakaran Murugesan[2], John M. Post[2], Vitor Mendes[3], Zhe Wang[4#], Navid Nahiyaan[4], Sasha L. Lynch[1], Stephen Thompson[2], Simon R. Green[2], Peter C. Ray[2], Jeannine Hess[5], Christina Spry[5$], Anthony G. Coyne[5], Chris Abell[5], Helena I. M. Boshoff[6], Paul G. Wyatt[2], Kyu Y. Rhee[4], Tom L. Blundell[3], Clifton E. Barry III[1,6], Valerie Mizrahi[1*]

Pages: 7

Figures: 8

Tables: 0

FIGURES

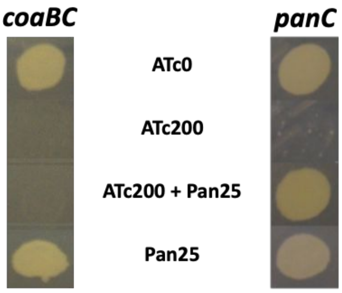

Figure S1: The effect of exogenous supplementation with pantothenate on the growth of CoaBC- and PanC-deficient Mtb. ATc – anhydrotetracycline (ng/mL); Pan25 – pantothenate (25 µg/mL).

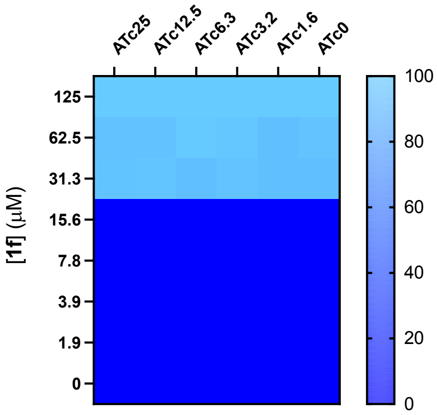

Figure S2: ATc has no effect on the susceptibility of Mtb H37RvMA to compound 1f when assessed at low cell density (OD_{600}=0.2).

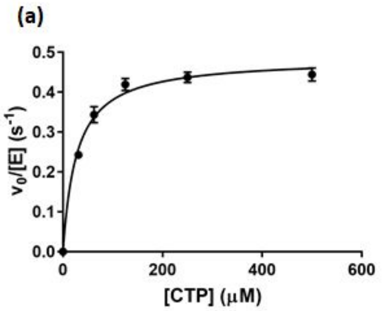

Figure S3: Mtb CoaBC saturation curve fit for Michaelis-Menten model. (a) CTP saturation curve. PPA and L-cysteine concentrations were fixed at 125 and 500 μM, respectively, while the CTP concentration was varied (31.25, 62.5, 125, 250 and 500 μM). (b) PPA saturation curve. CTP and L-cysteine concentrations were fixed at 125 and 500 μM, respectively, while the PPA concentration was varied (31.25, 62.5, 125, 250 and 500 μM). (c) L-cysteine saturation curve. CTP and PPA concentrations were fixed at 125 μM while the L-cysteine concentration was varied (31.25, 62.5, 93.75, 125, and 250 μM).

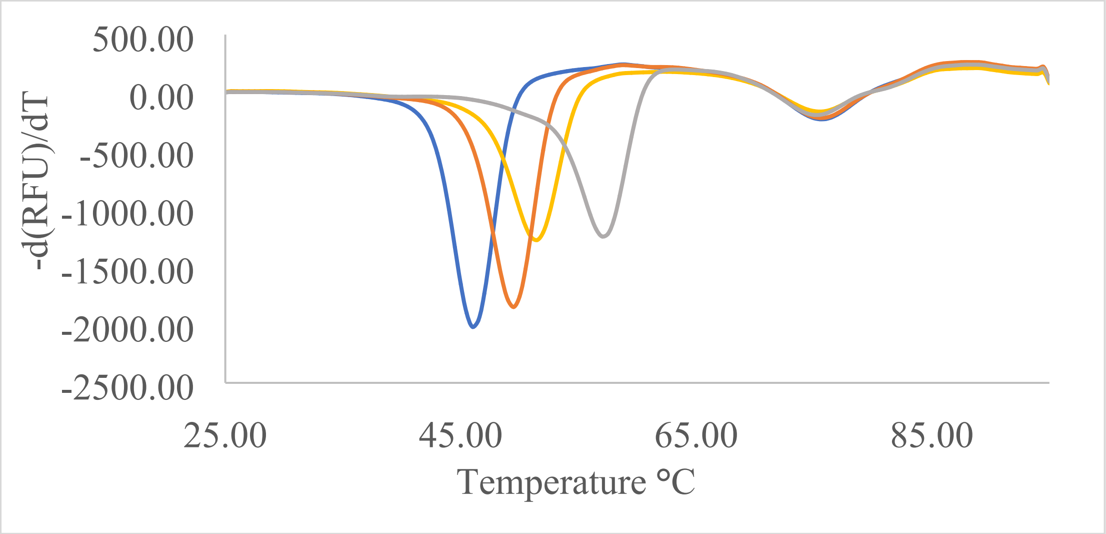

Figure S4: DSF melting profile of CoaBC. The blue line represents the melting profile of CoaBC without the addition of ligands, the orange line in the presence of PPA, the yellow line in the presence of CTP and the grey line in the presence of CTP and PPA.

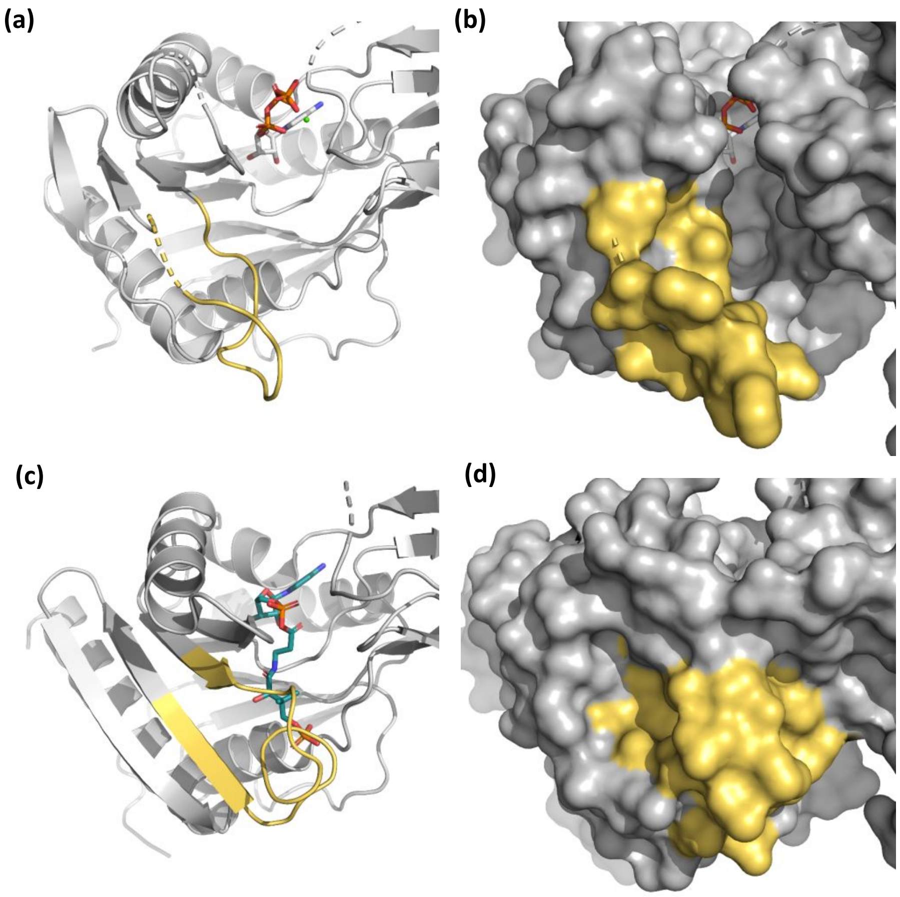

Figure S5: Movement of a flexible loop covering the PPA binding site. The protein region in yellow covers residues 361-378 in *M. smegmatis* CoaB (a-b) and residues 352-370 in *E. coli* CoaB (c-d) and corresponds to a loop that covers the PPA binding site upon PPA binding. (a-b) Loop in open conformation observed in *M. smegmatis* CoaB (PDB: 6TH2) with CTP and calcium bound and in closed conformation (c-d) as observed in *E. coli* CoaB after PPA binding (PDB: 1U7Z) with the reaction intermediate 4'-phosphopantothenoyl-CMP bound.

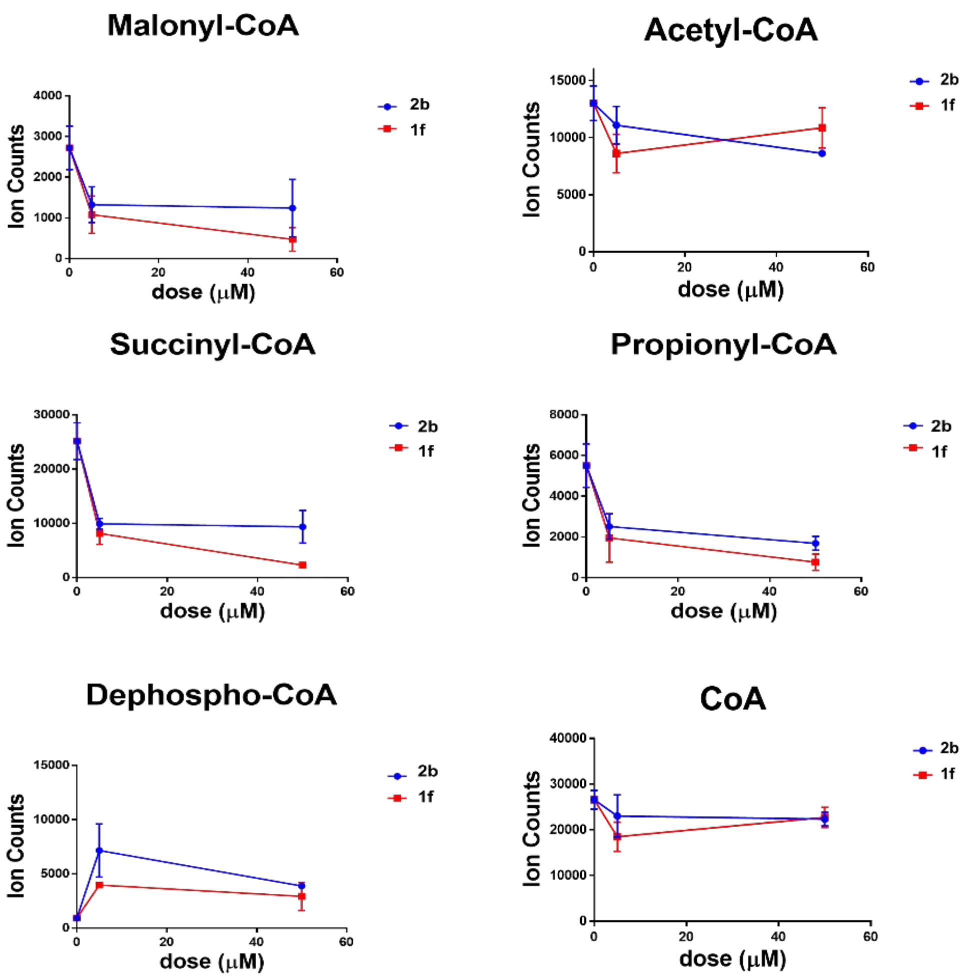

Figure S6: Impact of exposure of Mtb H37Rv to 1f and 2b on acyl-CoA species and CoA pathway metabolites.

(a)

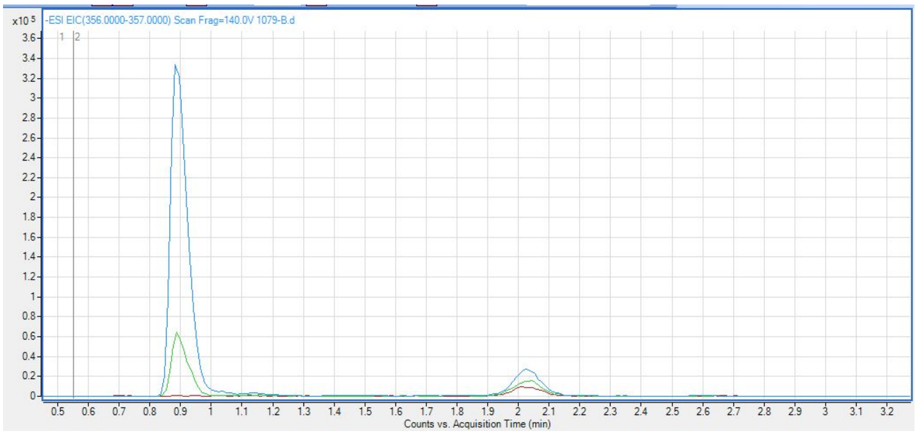

(b)

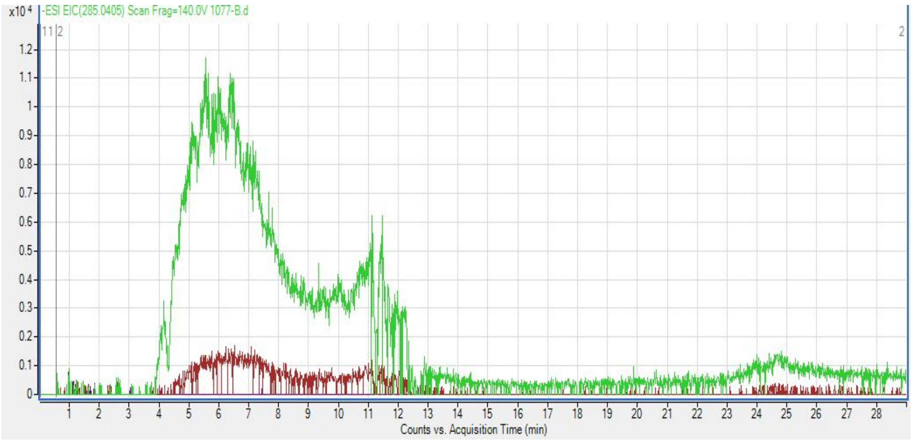

Figure S7: Total intracellular accumulation of (a) compound 1f (MW = 357.81 g/mol) and (b) 2b (MW = 286.24 g/mol). (a) the green and blue lines represent bacteria incubated in the presence of 5 µM and 50 µM **1f**, respectively; (b) the red and green lines represent bacteria incubated in the presence of 5 µM and 50 µM **2b**, respectively. X-axis is time (minutes), Y-axis is ion counts. Data are representative of 3 independent replicates.

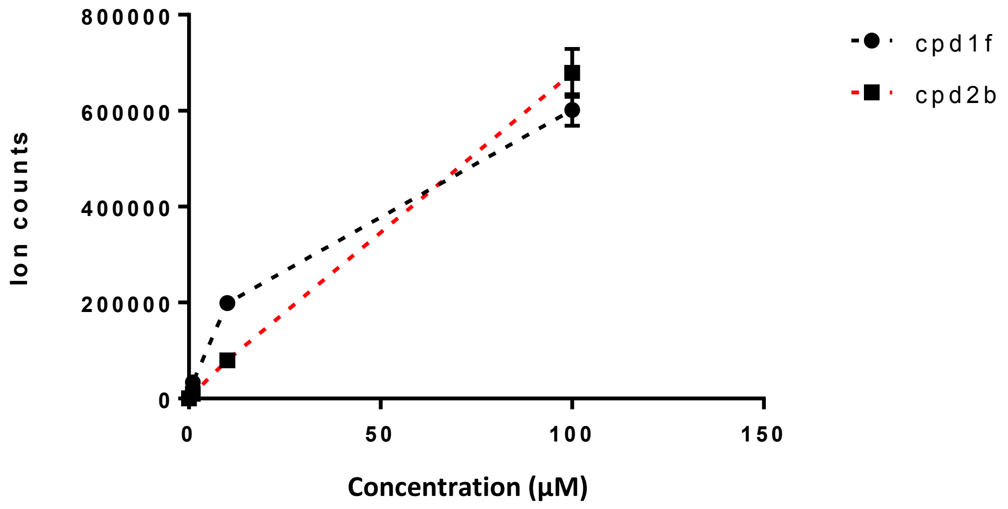

Figure S8: Standard curves showing comparable ionisation efficiencies of compounds 1f and 2b spiked into bacterial extracts. Internal standards are routinely included with each sample run, and data are normalised to sample protein biomass.
